# Machine learning and feature extraction for rapid antimicrobial resistance prediction of *Acinetobacter baumannii* from whole-genome sequencing data

**DOI:** 10.3389/fmicb.2023.1320312

**Published:** 2024-01-11

**Authors:** Yue Gao, Henan Li, Chunjiang Zhao, Shuguang Li, Guankun Yin, Hui Wang

**Affiliations:** ^1^Institute of Medical Technology, Peking University Health Science Center, Beijing, China; ^2^Department of Clinical Laboratory, Peking University People's Hospital, Beijing, China

**Keywords:** *Acinetobacter baumannii*, whole-genome sequencing, antimicrobial resistance prediction, machine learning, k-mer

## Abstract

**Background:**

Whole-genome sequencing (WGS) has contributed significantly to advancements in machine learning methods for predicting antimicrobial resistance (AMR). However, the comparisons of different methods for AMR prediction without requiring prior knowledge of resistance remains to be conducted.

**Methods:**

We aimed to predict the minimum inhibitory concentrations (MICs) of 13 antimicrobial agents against *Acinetobacter baumannii* using three machine learning algorithms (random forest, support vector machine, and XGBoost) combined with k-mer features extracted from WGS data.

**Results:**

A cohort of 339 isolates was used for model construction. The average essential agreement and category agreement of the best models exceeded 90.90% (95%CI, 89.03–92.77%) and 95.29% (95%CI, 94.91–95.67%), respectively; the exceptions being levofloxacin, minocycline and imipenem. The very major error rates ranged from 0.0 to 5.71%. We applied feature selection pipelines to extract the top-ranked 11-mers to optimise training time and computing resources. This approach slightly improved the prediction performance and enabled us to obtain prediction results within 10 min. Notably, when employing these top-ranked 11-mers in an independent test dataset (120 isolates), we achieved an average accuracy of 0.96.

**Conclusion:**

Our study is the first to demonstrate that AMR prediction for *A. baumannii* using machine learning methods based on k-mer features has competitive performance over traditional workflows; hence, sequence-based AMR prediction and its application could be further promoted. The k-mer-based workflow developed in this study demonstrated high recall/sensitivity and specificity, making it a dependable tool for MIC prediction in clinical settings.

## 1 Introduction

Multidrug-resistant (MDR) *Acinetobacter baumannii* strains have been reported by the World Health Organization as one of the most serious global threats to human health. Approximately 45% of *A. baumannii* isolates worldwide are classified as MDR, showcasing a notable ability for the acquisition of antimicrobial resistance (AMR) determinants and clonal transmission (De Oliveira et al., 2020); in China, this rate has reached 70%, from 2011 to 2020 according to the most recent studies (Chen et al., 2022; Yan et al., 2022; Chen et al., 2023). Few antibiotic agents show activity against *A. baumannii*, which significantly increases the mortality rates of infected individuals (Hu et al., 2023). From a clinical perspective, resolving MDR *A. baumannii* infection, reducing the time for optimal antimicrobial therapy, and preventing its further spread are critical for improving patient outcomes (Antimicrobial Resistance, 2022); thus, rapid and accurate AMR diagnostic methods are urgently required.

Traditional antimicrobial susceptibility testing (AST) relies on microbial culture, which is time-consuming (at least 36 h) and only viable for cultivable bacteria (Opota et al., 2015). Recently, modern molecular assays have accelerated the AST process by reducing the number of repetitive culture steps. With the growing accessibility of bacterial whole-genome sequencing (WGS), the cost and time of sequencing have decreased, and accuracy has improved (Nguyen et al., 2023). WGS provides high-resolution for phenotyping and surveillance, enabling the assessment of phylogenetic relationships, investigations of outbreaks, and predictions for virulence and epidemicity (Rodrigues et al., 2021; Hernandez-Gonzalez et al., 2022). By analysing, retrieving, and re-analysing existing sequences, WGS for AMR provides full insight into resistance genes present in a larger number of strains and characterisation of mutations that might confer resistance (Cooper et al., 2020). The increase in sequence data for well-characterised isolates has facilitated the development of computational frameworks for automated AMR prediction using machine-learning methods (Humphries et al., 2023). The most common approach for predicting AMR phenotypes from WGS data relies on meticulously curated AMR databases and precisely defined AMR genes (Stoesser et al., 2013; Bradley et al., 2015; Pesesky et al., 2016; Eyre et al., 2017). Pre-existing knowledge is indispensable in obtaining reliable results (Maguire et al., 2019). A limited number of studies have focused on applying WGS and AMR phenotype data without prior information to train machine-learning models for AMR prediction of non-typhoidal *Salmonella*, *Escherichia coli*, and *Klebsiella pneumoniae* (Nguyen et al., 2018; Maguire et al., 2019; Nguyen et al., 2019; Humphries et al., 2023; Wang et al., 2023). Although this reference-free approach necessitates a substantial volume of genomes, it is unbiased and thus facilitates the detection of novel genomic features implicated in AMR (Drouin et al., 2016). For other ESKAPE pathogens (Rice, 2008), comparisons of different machine-learning methods for AMR prediction based on WGS data without requiring prior knowledge of mechanisms or mutations for resistance remain to be performed.

To this end, our study aimed to evaluate the random forest (RF), support vector machine (SVM), and extreme gradient boosting (XGBoost) models to predict the phenotypic susceptibility of *A. baumannii* to 13 antimicrobial agents. We used nucleotide 11-mers extracted from each genome and the minimum inhibitory concentrations (MICs) of each antimicrobial agent as input features to train the models. Then, a feature selection pipeline was developed to identify significant AMR phenotype determinants from the models without any prior information. In many cases, the models that used only a few important features selected by our feature selection pipeline obtained better prediction outcomes, with lower computational requirements, less computational resources needed, and significantly shorter training time than those using all 11-mers features. Application of the top-ranked 11-mers models to an additional 120 isolates used as an independent test dataset also achieved good performance. In our new scheme, we obtained the predicted MICs of 13 antimicrobial agents against *A. baumannii* in 10 min and showed competitive performance compared to traditional workflows.

## 2 Materials and methods

The present study was reviewed and approved by the Research Ethics Board at Peking University People’s Hospital (Beijing, China). Written informed consent was not required because the medical records and patient information were anonymously reviewed and collected.

### 2.1 Bacterial isolates and AST

We used data from the Chinese Antimicrobial Resistance Surveillance of Nosocomial Infections (CARES, 2016 and 2018, involving 14 teaching hospitals from nine central cities of China) (Liu et al., 2020; Yin et al., 2021). A total of 339 non-duplicate *A. baumannii* isolates collected mainly from bloodstream infections and hospital-acquired pneumonia were included in this study. To further evaluate the developed model, we utilised an additional testing dataset that included 60 *A. baumannii* isolates collected randomly from the Chinese Meropenem Surveillance Study (CMSS) (2016 and 2018), Peking University People’s Hospital (PKUPH) (2017 to 2019), and CARES (2020 and 2021), which involved 11 central cities of China. For more extensive data from public sources, we also screened the PAThosystems Resource Integration Center (PATRIC) database (Wattam et al., 2017) and collected 60 *A. baumannii* isolates with raw sequencing data and the most extensive AST data based on the 13 antimicrobial agents that we trained. All 120 isolates were completely independent of the dataset used for model construction. AST was performed on all *A. baumannii* isolates using the agar dilution method for 11 antimicrobial agents, including imipenem (IPM), meropenem (MEM), cefepime (FEP), ceftazidime (CAZ), cefoperazone-sulbactam (CSL), piperacillin-tazobactam (TZP), amikacin (AMK), ciprofloxacin (CIP), levofloxacin (LVX), minocycline (MIN), and trimethoprim-sulfamethoxazole (SXT). The MICs of colistin (CST) and tigecycline (TGC) were determined by the broth microdilution method. The susceptible, intermediate, and resistant categories were determined according to the Clinical and Laboratory Standards Institute guidelines 2022-M100.^1^ Susceptibility to CSL was interpreted based on the MICs of cefoperazone for *Enterobacteriaceae* (Ku and Yu, 2021), and susceptibility to TGC was interpreted according to the US Food and Drug Administration (FDA) criteria as susceptible: ≤ 2 mg L^−1^, resistant: ≥ 8 mg L^−1^ (Jo and Ko, 2021). *Escherichia coli* ATCC 25922 and *Pseudomonas aeruginosa* ATCC 27853 were used as quality control strains.

### 2.2 WGS and dataset preparation

Genomic DNA was extracted from pelleted bacteria using DNA purification kits (QIAGEN, Hilden, Germany), and paired-end sequencing was performed using Illumina NovaSeq 6,000 (Illumina Inc., CA, USA). Readfq V8^2^ was used to evaluate the sequencing reading quality according to the Phred scoring system. After using fastp (version 0.20.1) (Chen et al., 2018) and SPAdes (version 3.13.0) (Prjibelski et al., 2020) to quality-trim and assemble the resulting FASTQ files, we obtained clean whole-genome sequences and used QUAST (version 5.10.0) (Mikheenko et al., 2018) for genome assembly evaluation and comparison. According to the Institut Pasteur MLST protocols and database,^3^ multilocus sequence typing (MLST) was characterised for each *A. baumannii* isolate. We utilised KMC 3 (Kokot et al., 2017) to extract 11-mers from FASTQ files. In this study, the parameter k = 11 was used due to computational memory limitations. We used Pandas (version 1.0.5) (Wes, 2011) to build a matrix in which the k-mer counts in the columns were treated as unique features for each genome in a row. Rather than directly dividing the genomes into susceptible or non-susceptible, MIC labels corresponding to each genome were converted to one-hot codes using LabelBinarizer (scikit-learn, version 0.23.1) (Pedregosa et al., 2011). This allowed us to employ the OneVsRest classifier, which offers high interpretability and the opportunity to gain insights into each class by inspecting its corresponding categories. Finally, the two matrices were used for each antibiotic as input data for the subsequent machine-learning process.

### 2.3 Model generation

All data were shuffled into 80% training and 20% testing datasets. To select the best model for each antibiotic, we tested RF (Breiman, 2001), SVM with three kernels (linear, polynomial, and RBF) (Cortes and Vapnik, 1995), and XGBoost (Chen et al., 2016). For XGBoost, the Python implementation (version 1.4.0) was used during the training phase; for the remaining methods, we utilised Scikit-learn (version 0.23.1). The accuracy and recall/sensitivity of the models were tested using ten-fold Stratified Shuffle-Split Cross-Validation. For each cross-validation, the training and testing data partition was randomly determined. After ten rounds (folds) of training, the best-performing model was selected, and the final outcome was determined by calculating the average of the results.

The comparison results can be found in the Supplementary Materials. Since RF outperformed the other methods with reasonable computational complexity, we used the built-in feature importance function to obtain the importance scores of each feature. The top-500 feature importance scores were then arranged to generate an overall importance feature for each antibiotic. Subsequently, the top-ranked 11-mers features were adopted for independent model training and validation, as described above. The analysis scripts are available at https://github.com/yuegao-pkuhsc/aba_mic_prediction.

### 2.4 Model evaluation and interpretation of results

According to FDA requirements for automated MIC measuring device standards (Administration, U.S.F.a.D, 2009), the models’ accuracy was measured by their capability to predict the correct MIC within ±1 of the two-fold dilution step of the laboratory-derived MIC. Termed essential agreement (EA) and category agreement (CA), receiver operating characteristic (ROC) curves, and area under the curve (AUC) values were also used to judge the predictive performance of the models. For the final classification results based on clinical breakpoints, the recall/sensitivity, specificity, positive predictive value (PPV), negative predictive value (NPV), major error (ME), and very major error (VME) were also calculated to evaluate model performance as follows: Recall/Sensitivity = TP/(TP + FN); Specificity = TN/(TN + FP); PPV = TP/(TP + FP); NPV = TN/(TN + FN); ME = FP/(TN + FP); VME = FN/(TP + FN), where TP, FN, TN, and FP represent true positives, false negatives, true negatives, and false positives, respectively (Wang et al., 2023).

## 3 Results

### 3.1 Overview of genome and AST features

**Figure 1:**
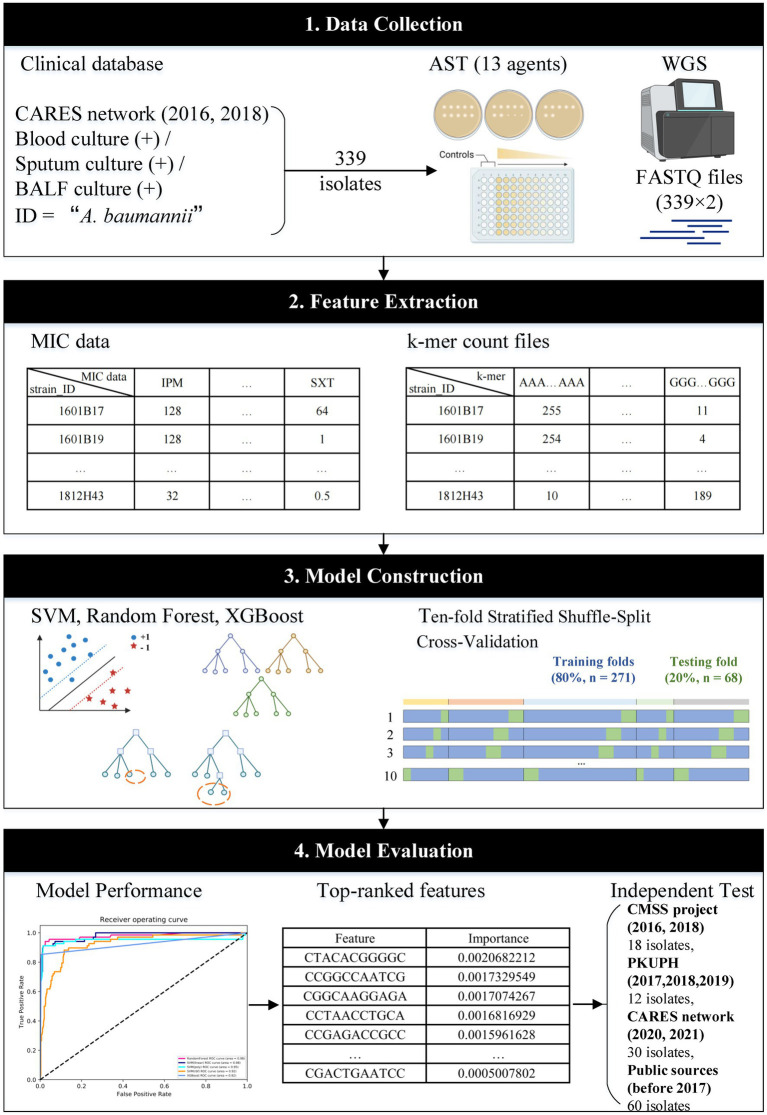
Workflow of this study. MIC data and k-mer count files of 339 isolates were used to train and cross-validate the three main machine-learning algorithms. The built-in feature importance function was utilised to rank the features for independent tests. The workflow was created with BioRender.com.

**Figure 2:**
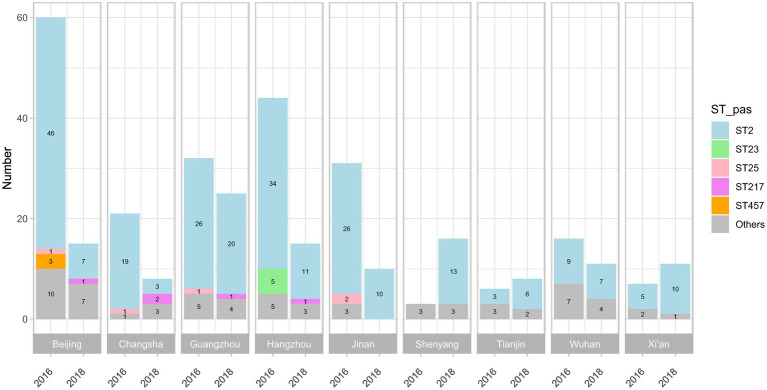
Sequence types (STs) of the 339 isolates collected in 2016 and 2018, according to the Institut Pasteur MLST scheme. A total of 40 STs were identified, and the STs of 24 isolates were unknown; ST2, ST23, and ST25 were the most abundant in all isolates.

The workflow of this study is illustrated in Figure 1. The *A. baumannii* strains were mainly isolated from bloodstream infections (109/339, 32.2%) and hospital-acquired pneumonia cases (230/339, 67.8%) from 14 teaching hospitals in nine central cities, which represents four regions of China: the Northern Region (including Beijing, Tianjin, Jinan, and Shenyang); Eastern Region (Hangzhou); Central Region (Xi’an, Wuhan, and Changsha); and Southern Region (Guangzhou). After using WGS to obtain the DNA sequences of all isolates, over 90% of reads per genome surpassed the standard threshold (Q30), indicating the high quality of the sequencing reads. All assembly statistics are shown in Supplementary Table S1. Forty different sequence types (STs) were then identified in the model construction dataset, with ST2 (255/339, 75.2%) being the most abundant. The geographical and temporal distributions are shown in Figure 2. For the independent test dataset, 120 isolates were randomly collected from CMSS (2016 and 2018, 18 isolates), PKUPH (2017 to 2019, 12 isolates), CARES (2020 and 2021, 30 isolates), and public data sources (before 2017, 60 isolates). These isolates had significant differences in geographic and temporal distribution compared to the 339 isolates used for model construction to simulate the application of the abovementioned models for AMR surveillance. However, ST2 was still the most abundant (84/120, 70.0%), which is in line with previous studies that show that ST2 is widely spread globally and represents the predominant clone of MDR *A. baumannii* (Hamidian and Nigro, 2019; Li et al., 2023). This is especially true for China, where ST2 affects a high proportion of hospitalised patients and contributes to significant mortality rates (Yu et al., 2021; Liu et al., 2022; Wei et al., 2023). Specimen collection, sampling location, accession number, and other details of all 459 isolates can be found in Supplementary Table S2. The 11-mer features derived from the genome sequences of the model construction dataset (339 isolates) were used for machine learning. Theoretically, there are 4^11^ = 4,194,304 features. Upon excluding the 11-mer features absent from our dataset, the overall feature count amounted to 2,097,076.

The isolates (*n* = 339) were tested for resistance to 13 antimicrobial agents using agar dilution and broth microdilution methods (TGC and CST). Of all antimicrobial agents, only data for four SXT were missing. Considering the AST phenotypes, the percentages of non-susceptibility were found to be 80.83, 80.83, 4.72, 6.19, 82.01, 81.12, 79.94, 82.01, 70.50, 81.71, 80.24, 56.34, and 66.57% for IPM, MEM, CST, TGC, FEP, CAZ, CSL, TZP, AMK, CIP, LVX, MIN, and SXT, respectively. For the independent test dataset, 13 antimicrobial agents were also evaluated for the 60 isolates; for the other 60 isolates, MIC values were downloaded from the PATRIC database. Detailed AST results of all 459 isolates are shown in Supplementary Table S3. The model construction dataset was split randomly into training (*n* = 271) and testing (*n* = 68) sets using an 8:2 ratio, which was guided by the principles of stratified sampling. To guarantee that all MICs of all antimicrobial agents could be effectively trained as separate categories within the training set, we used the Stratified ShuffleSplit software to generate stratified randomised folds while maintaining the proportion of samples for each MIC level.

### 3.2 Model performance for the MIC prediction of *Acinetobacter baumannii*

After performing WGS and AST on all isolates to obtain 11-mer features and MIC data, the overall performance of the RF, SVM (linear, polynomial, and RBF), and XGBoost models was evaluated based on the two matrices obtained for each antimicrobial agent. ROC curves and AUC values indicate the efficacy of machine-learning models (Fawcett, 2006). In this study, RF performed better than the other models, with the average cross-validation results for the 13 antimicrobial agents were all ≥0.945 (Figure 3A; Supplementary Figures S1–S13; Supplementary Table S4).

**Figure 3:**
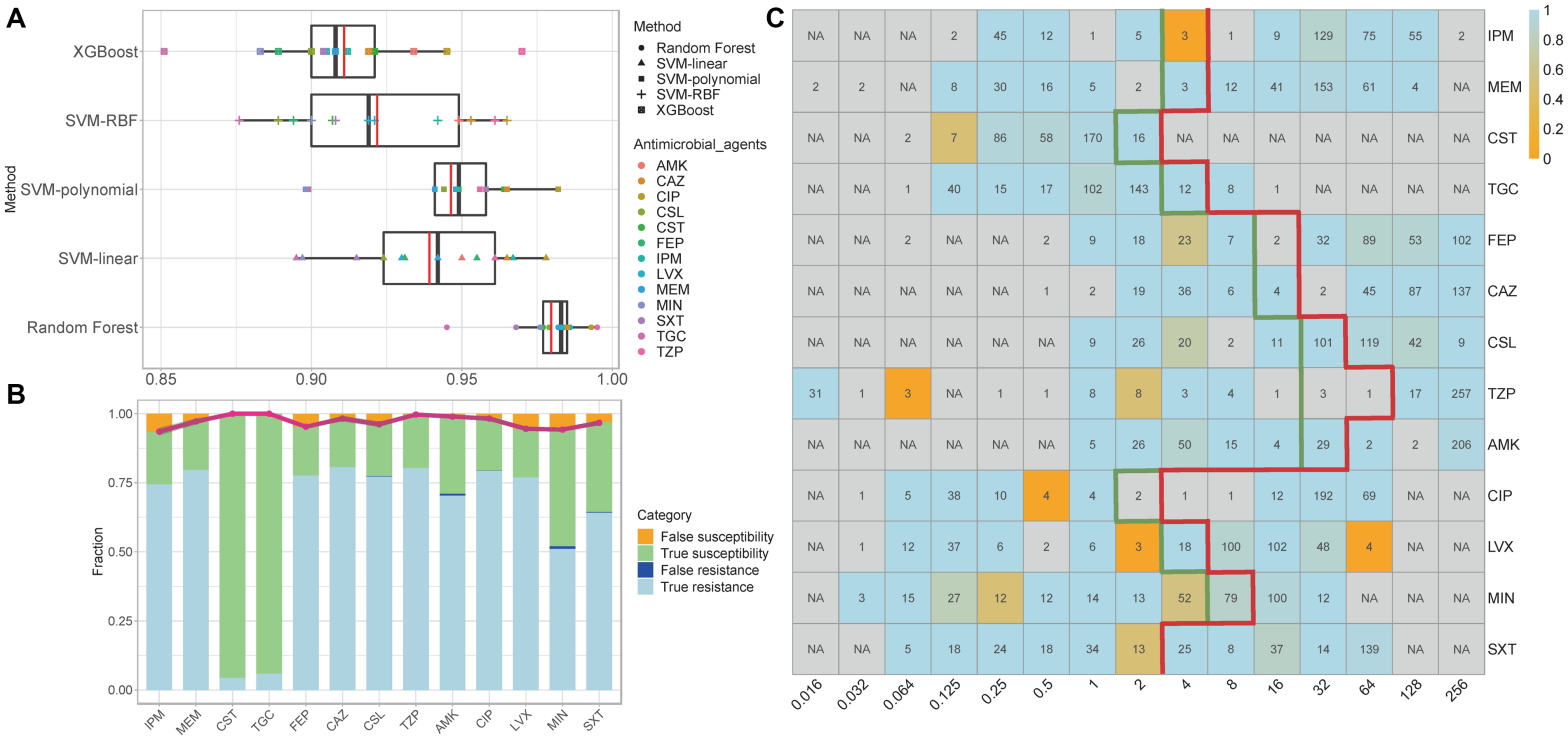
**(A)** Median and mean AUC values of five models for the 13 antimicrobial agents. In each box plot, the whiskers represent the maximum and minimum. The boxes represent the first and the third quartiles. The black line represents the median, and the red line represents the mean. **(B)** Comparison of prediction results of the best models for the 13 antimicrobial agents. The bars show the rate of true resistance, false resistance, true susceptibility, and false susceptibility. The broken line depicts the predictive accuracy. The grey shadow surrounding the line represents the 95% confidence interval. **(C)** Average accuracies within ±1 in the two-fold dilution step. The values shown in each cell are the number of genomes with that MIC for a given antibiotic; ‘NA’ indicates no isolates under the specific MIC level. The thick green line indicates the MIC breakpoints for ‘susceptible’, and the thick red line indicates the MIC breakpoints for “resistant.” The accuracy is depicted by colour, with light blue being the most accurate and orange being the least accurate. IPM, imipenem; MEM, meropenem; CST, colistin; TGC, tigecycline; FEP, cefepime; CAZ, ceftazidime; CSL, cefoperazone-sulbactam; TZP, piperacillin-tazobactam; AMK, amikacin; CIP, ciprofloxacin; LVX, levofloxacin; MIN, minocycline; SXT, trimethoprim-sulfamethoxazole.

The specific best prediction and standard results for each antimicrobial agent are shown in Supplementary Tables S5–S30. We used the average results and 95% confidence interval (CI) of ten cross-validations of EA, CA, recall/sensitivity, specificity, PPV, NPV, ME, and VME to measure the best model’s performance (Table 1). Although the data structure and volume of some antimicrobial agents (such as CST and TGC) were unbalanced, the CAs for all antimicrobial agents exceeded 93%. With the exception of MIN, the EAs for all antimicrobial agents exceeded 90%, which met the acceptable percentage of the FDA standards (Administration, U.S.F.a.D, 2009). Figure 3B compares the prediction results of the best models, and Figure 3C shows the average accuracy of the best models for each MIC of the 13 antimicrobial agents. The models tended to have lower accuracies when few genomes represented the MIC. Overall, the models exhibited robustness for both non-susceptible and susceptible MICs. Furthermore, the recall/sensitivity was >91% and the specificity was >97% for all antimicrobial agents. MEs were identified as susceptible genomes to which non-susceptible MICs were inaccurately assigned, whereas VMEs referred to non-susceptible genomes to which susceptible MICs were inaccurately assigned using the machine-learning models. The standards stipulated by the FDA for automated systems prescribe an ME rate of ≤3% and an upper confidence limit of ≤5.96% (at 95% confidence level) for VME rates. Nine of the 13 antimicrobial agents (MEM, CST, TGC, CAZ, CSL, TZP, AMK, CIP, and SXT) had acceptable ME and VME rates (Table 1). Since the RF model demonstrated resilience against diverse mechanisms of resistance, FEP had slightly higher VME rates, while MEM, LVX and MIN had lower accuracies, probably because of MICs less than, equal to, or greater than a certain value, which represent a range of MICs defined as discrete values in the machine learning process. We also noticed that the ME and VME rates of CST and TGC were as low as 0%, this might be due to the fact that naturally collected isolates resistant to tigecycline and colistin are relatively infrequent in clinical settings and even public sources, resulting in a dataset with a lesser quantity of resistant isolates. We expect the improvements in both ME and VME rates with a corresponding expansion in the collection of genomes and a greater balance of the dataset.

**Table 1:** Performance of the best models to predict *A. baumannii* susceptibility or non-susceptibility for 13 antimicrobial agents.

Antimicrobial agents	Recall/Sensitivity [95% CI]	Specificity [95% CI]	NPV [95% CI]	PPV [95% CI]	VME [95% CI]	ME [95% CI]	EA [95% CI]	CA [95% CI]
Imipenem	92.00% [90.30%; 93.70%]	100.00% [100.00%; 100.00%]	75.25% [71.00%; 79.50%]	100.00% [100.00%; 100.00%]	8.00% [6.30%; 9.70%]	0.00% [0.00%; 0.00%]	93.09% [91.48%; 94.70%]	93.53% [92.16%; 94.90%]
Meropenem	96.61% [95.28%; 97.94%]	100.00% [100.00%; 100.00%]	86.91% [82.29%; 91.53%]	100.00% [100.00%; 100.00%]	3.39% [2.06%; 4.72%]	0.00% [0.00%; 0.00%]	95.00% [93.56%; 96.44%]	97.21% [96.12%; 98.30%]
Colistin	100.00% [100.00%;100.00%]	100.00% [100.00%; 100.00%]	100.00% [100.00%;100.00%]	100.00% [100.00%; 100.00%]	0.00% [0.00%; 0.00%]	0.00% [0.00%; 0.00%]	93.09% [91.73%; 94.45%]	100.00% [100.00%; 100.00%]
Tigecycline	100.00% [100.00%; 100.00%]	100.00% [100.00%; 100.00%]	100.00% [100.00%;100.00]	100.00% [100.00%; 100.00%]	0.00% [0.00%; 0.00%]	0.00% [0.00%; 0.00%]	95.59% [93.98%; 97.20%]	100.00% [100.00%; 100.00%]
Cefepime	94.29% [93.82%; 94.76%]	100.00% [100.00%; 100.00%]	79.00% [77.69%; 80.31%]	100.00% [100.00%; 100.00%]	5.71% [5.24%; 6.18%]	0.00% [0.00%; 0.00%]	92.50% [91.41%; 93.59%]	95.29% [94.91%; 95.67%]
Ceftazidime	97.86% [96.60%; 99.12%]	100.00% [100.00%; 100.00%]	91.49% [86.79%; 96.19%]	100.00% [100.00%; 100.00%]	2.14% [0.88%; 3.40%]	0.00% [0.00%; 0.00%]	97.14% [96.26%; 98.02%]	98.24% [97.21%; 99.27%]
Cefoperazone-sulbactam	95.45% [94.23%; 96.67%]	99.23% [97.72%; 100.00%]	84.15% [80.51%; 87.79%]	99.81% [99.45%; 100.00%]	4.55% [3.33%; 5.77%]	0.77% [0.00%; 2.28%]	94.71% [93.41%; 96.01%]	96.18% [95.20%; 97.16%]
Piperacillin-tazobactam	99.63% [99.15%; 100.00%]	100.00% [100.00%; 100.00%]	98.57% [96.70%;100.00]	100.00% [100.00%; 100.00%]	0.37% [0.00%; 0.85%]	0.00% [0.00%; 0.00%]	98.36% [97.25%; 99.47%]	99.70% [99.31%; 100.00%]
Amikacin	99.58% [99.04%; 100.00%]	97.50% [95.87%; 99.13%]	99.02% [97.74%;100.00]	98.98% [98.31%; 99.65%]	0.42% [0.00%; 0.96%]	2.5% [0.87%; 4.13%]	93.97% [92.12%; 95.82%]	98.97% [98.35%; 99.59%]
Ciprofloxacin	98.00% [97.01%; 98.99%]	99.23% [97.72%; 100.00%]	92.45% [89.03%; 95.87%]	99.82% [99.46%; 100.00%]	2.00% [1.01%; 2.99%]	0.77% [0.00%; 2.28%]	97.35% [96.63%; 98.07%]	98.24% [97.40%; 99.08%]
Levofloxacin	93.39% [92.48%; 94.30%]	100.00% [100.00%; 100.00%]	76.28% [73.72%; 78.84%]	100.00% [100.00%; 100.00%]	6.61% [5.70%; 7.52%]	0.00% [0.00%; 0.00%]	93.82% [92.79%; 94.85%]	94.54% [93.78%; 95.30%]
Minocycline	91.32% [89.98%; 92.66%]	98.00% [96.93%; 99.07%]	89.98% [88.62%; 91.34%]	98.34% [97.45%; 99.23%]	8.68% [7.34%; 10.02%]	2.00% [0.93%; 3.07%]	88.24% [86.18%; 90.30%]	94.26% [93.74%; 94.78%]
Trimethoprim-sulfamethoxazole	95.51% [94.04%; 96.98%]	99.13% [97.99%; 100.00%]	91.93% [89.54%; 94.32%]	99.54% [98.94%; 100.00%]	4.49% [3.02%; 5.96%]	0.87% [0.00%; 2.01%]	90.90% [89.03%; 92.77%]	96.72% [95.76%; 97.68%]

CI, confidence interval.

### 3.3 Top-ranked 11-mers as predictive features

The developed models exhibited predictive capabilities. However, the presence of high-dimensional feature vectors may potentially impact machine learning performance and increase execution time (Wang et al., 2023). We further analysed the best models of the 13 antimicrobial agents and used the feature and importance function estimator integrated within RF and SVM-linear to summarise and rank the importance of the 11-mers features. The top-ranked 11-mers features were selected for each antimicrobial agent according to the importance scores (Supplementary Table S31). Subsequently, RF was applied to construct prediction models for each agent utilising the MIC dataset and top-ranked 11-mer features.

**Figure 4:**
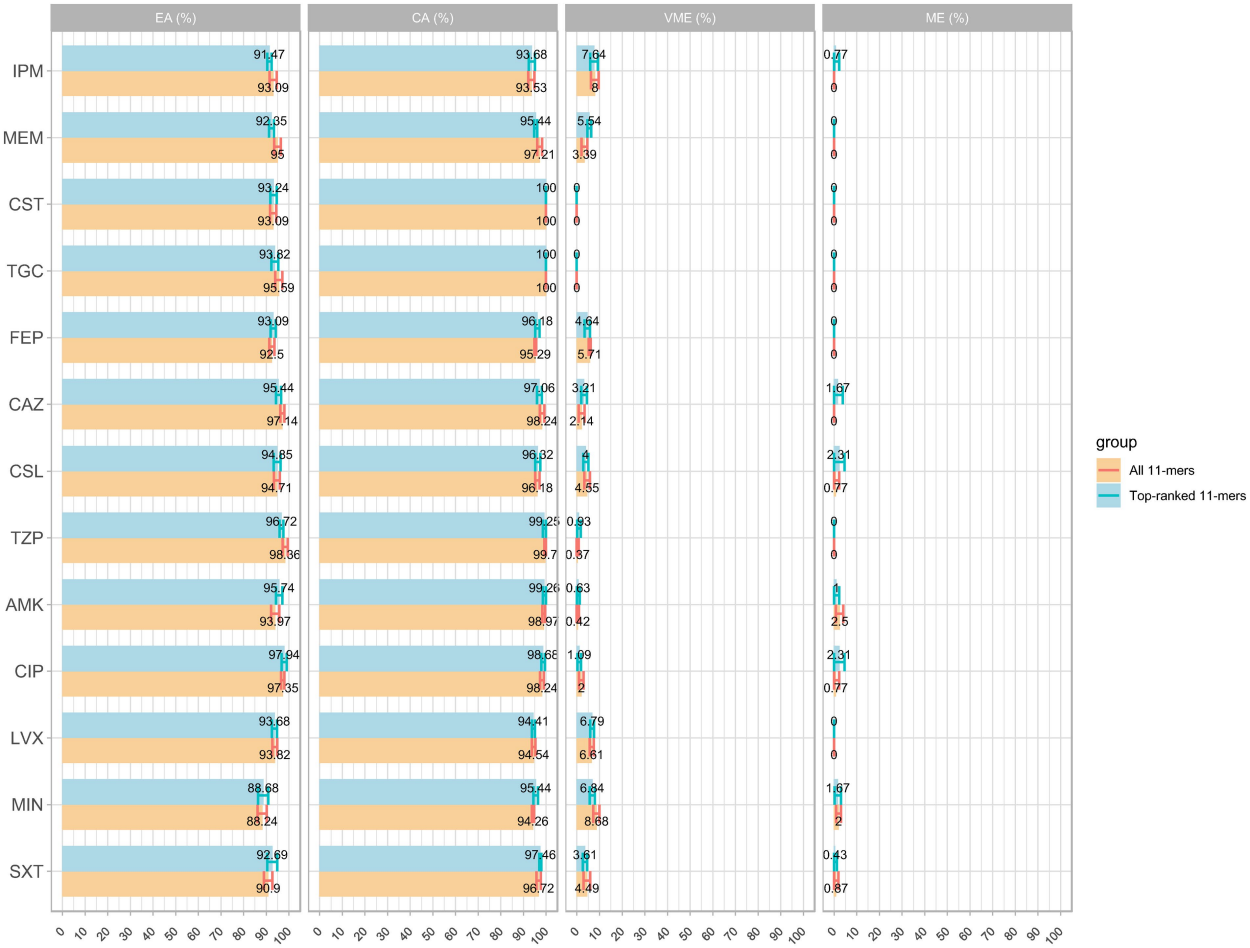
Comparison of MIC prediction based on all k-mers versus top-ranked 11-mers: the average results of EA, CA, VME, and ME with 95% confidence interval.

The models using the top-ranked 11-mers features demonstrated good performance, similar to those using over two million 11-mers features as input, with all average AUC values were > 0.9 (figures not shown). A detailed comparison of the means and 95% CIs for EA, CA, VME, and ME is shown in Figure 4. Unexpectedly, even a small improvement was observed using the top-ranked 11-mers features. The VME rate of FEP was lower than that observed when using all 11-mers, to the extent that it satisfied FDA standards; The average EA and CA of the model using the top-ranked 11-mers were 93.82 and 97.17%, respectively, while the model using all 11-mers showed averages of 94.14% for EA and 97.14% for CA. Given that the top-ranked 11-mer features performed well on the 339 isolates collected in 2016 and 2018, an additional dataset comprising 120 isolates was used as an independent test. The models using the top-ranked 11-mers achieved an average prediction accuracy of 0.96 across the 13 antimicrobial agents within ±1 in the two-fold dilution step, indicating the robust performance of these models. More detailed information can be found in Supplementary Table S32.

These findings imply that the top-ranked 11-mers are crucial components for MIC prediction and could potentially outperform models that utilise all 11-mers features. Altogether, the top-ranked 11-mers models are useful for improving MIC prediction by significantly optimising the training time and computing resources and may provide valuable insights for future research.

## 4 Discussion

**Figure 5:**
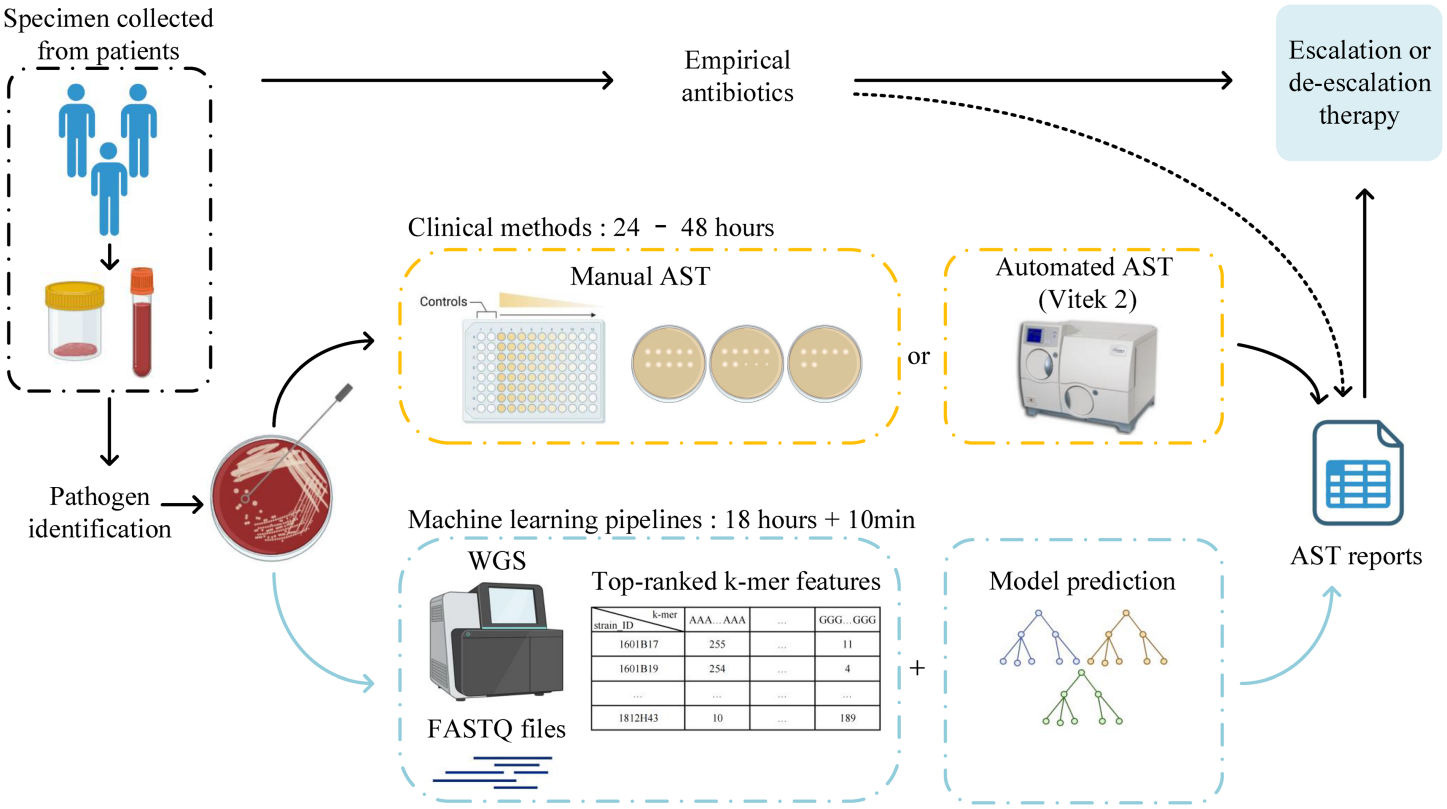
Traditional workflow for clinical laboratories to obtain AST reports from the bedside, which takes at least 3 to 4 days due to the reliance on bacterial culture. The new scheme of this study can obtain AST reports earlier by integrating WGS combined with machine-learning models into the traditional workflow. The workflow was created with BioRender.com.

In the traditional workflow, after collecting specimens from the bedside, culturing, bacterial identification, and AST, at least three to four days are required to obtain the AMR phenotypes of *A. baumannii* isolates. The present study established MIC prediction models for 13 antimicrobial agents against *A. baumannii* isolates using different machine-learning methods combined with 11-mer features. The overall EAs and CAs reached 94.14 and 97.14%, respectively. By integrating WGS combined with our machine-learning models into the traditional workflow, we obtained AST reports at least six hours earlier than we would in routine clinical testing (Figure 5). Thus, this model exhibits the potential to assist the screening of *A. baumannii* isolates and provide clinicians with guidance before AST reports become available.

Methods of AMR prediction from bacterial genomes are mainly based on pre-existing genetic AMR determinants or conserved genes (Pesesky et al., 2016; Macesic et al., 2020; Nguyen et al., 2020). Several databases providing a large number of known AMR determinants are required for these methods, including the PATRIC (Wattam et al., 2017), Comprehensive Antibiotic Resistance Database (CARD) (McArthur et al., 2013), and ResFinder (Zankari et al., 2012). Although prior knowledge-based AMR prediction methods demonstrate high accuracy, their main limitation is that their effectiveness is restricted to situations in which the AMR mechanisms are known. Additionally, these methods show a lack of adaptability when it comes to incorporating updates from databases. Generally, these methods are premised on the belief that AMR arises from a single genetic factor. However, several studies have indicated that the presence of multiple co-existing mechanisms can alter the ultimate AMR phenotype (Vogwill et al., 2016; Porse et al., 2020). Furthermore, reliance solely on gene-centric methodologies can overlook pivotal mutations in non-coding areas, such as regulatory elements and promoter regions, potentially leading to inaccuracies in susceptibility prediction, including false-negative results (Nguyen et al., 2018). The methods reported in the present study can infer the AMR phenotype directly from FASTQ files using machine-learning techniques. Five machine learning models based on 11-mers were developed to predict MIC values for 13 antimicrobial agents. These models were thoroughly validated and tested using two separate datasets to ensure their reliability. This is the first study to use k-mer as a machine-learning input feature to predict the MICs of antimicrobial agents for *A. baumannii*. By comparing the average cross-validation results, RF performed better than SVM and XGBoost for the 13 antimicrobial agents, which may be due to the fact that the RF is a nonlinear machine learning algorithm, and its ability to make predictions based on complex rules derived from ensembled decision trees that incorporate 11-mer features (Wang et al., 2023). Since AMR can involve multiple genes simultaneously, RF’s approach can potentially provide better performance than other machine learning algorithms. By utilising advances in machine learning, one notable characteristic of the models in this study is that prior knowledge is no longer required; instead, the algorithms learn AMR knowledge from the data (Avershina et al., 2023). Although nucleotide k-mers can accurately predict AMR, as the value of k rises, the number of features exponentially grows until constrained by the genome size. Consequently, the presence of longer k-mers and a greater number of features may result in memory challenges during the training of machine-learning models. We built a feature selection pipeline to extract the top-ranked important k-mer features of *A. baumannii* isolates to make this process less time-consuming and more applicable. Models that use only a few top-ranked 11-mer features can predict resistance as accurate as models utilising all 11-mer features; the average EA and CA were 93.82 and 97.17%, respectively. To evaluate our models, we compared them with those from other relevant studies that are also based on k-mer features. In these published studies, for the phenotypic predictions of nontyphoidal *Salmonella*, the model developed by Maguire and colleagues exhibited a precision exceeding 91% (Maguire et al., 2019), while Nguyen and associates indicated an overall average accuracy of 95% within ±1 two-fold dilution step (Nguyen et al., 2019). As for the phenotypic prediction of *E. coli*, the model designed by Humphries and colleagues reached a categorical agreement of 97% (Humphries et al., 2023). Regarding the phenotypic prediction of *K. pneumoniae*, the model by Nguyen and associates demonstrated the overall accuracy of 92% (Nguyen et al., 2018). Although there are no current k-mer based prediction studies for *A. baumannii*, our results closely align with the outcomes of the prior published study based on pre-existing AMR mechanisms (Avershina et al., 2021), particularly for the agents such as AMK, CIP, LVX, and MEM. Furthermore, the top-ranked 11-mer models were externally validated using a cross-temporal, cross-regional and independent test dataset of 120 *A. baumannii* isolates, which also showed good performance. The comparison of the top-ranked 11-mers and all 11-mers models highlighted the significance of feature selection. The top-ranked 11-mers can be further mapped to the genome for identifying genes associated with AMR, which can also provide additional insights for researchers to discover new AMR mechanisms by analysing these features. Despite the distinct STs of the *A. baumannii* isolates examined in this study and the exclusion of strain evolution from consideration, the EAs and CAs for all antimicrobial agents exhibited generally high performance across the two kinds of models.

This study has some limitations. Data volume and structure limit the improvement of VMEs and MEs. Owing to disparity in the sample size of different MIC levels, the classifier could not fully grasp the MIC features when there were only a few isolates available. Given that the utilised dataset was sourced from the regular surveillance program, imbalances in the data were anticipated. Continuous updating of databases is required to avoid these extremes of distribution phenomena where there are only one or two isolates in some MICs. The public database also significantly overrepresents clinically important resistant bacterial strains in comparison to susceptible ones. Nevertheless, with ongoing advances in sequencing and high-throughput technologies, we foresee the databases becoming more enriched and balanced, leading to improved outcomes for machine learning applications (Sunuwar and Azad, 2021). The accurate prediction of MICs for *A. baumannii* isolates, particularly when dealing with strains at the extremes of the resistance spectrum, is complicated by potential underlying genetic variations; however, with the collection of more genomic data and the achievement of a more balanced dataset, we expect the ME and VME rates to decline and the accuracy of MICs and classification to further improve.

In conclusion, we present a machine-learning approach for AMR prediction in *A. baumannii*. The developed k-mer-based workflow demonstrated high recall/sensitivity and specificity, making it a reliable tool for MIC prediction in clinical settings. Machine-learning algorithms can decipher the mechanisms of AMR from DNA sequence data without the need for prior information. We extracted the top-ranked k-mer features of 13 antimicrobial agents that can be interpreted and exploited to obtain new information on resistance mechanisms. In combination with database improvements, our methodology could assist clinical laboratories in the rapid MIC prediction of *A. baumannii* to complement traditional AST. Importantly, the success of directly predicting MICs from a limited number of genomes indicates that it is viable to obtain susceptibility results without any prior information using machine-learning methods, an approach that can be extended to other pathogens.

## Data availability statement

The sequenced data of this study are available in NCBI Sequence Read Archive (SRA) with the BioProject PRJNA1014981.

## Author contributions

YG: Data curation, Investigation, Methodology, Software, Visualization, Writing – original draft, Writing – review & editing. HL: Data curation, Investigation, Writing – review & editing. CZ: Methodology, Software, Writing – review & editing. SL: Data curation, Writing – review & editing. GY: Data curation, Writing – review & editing. HW: Conceptualization, Project administration, Writing – review & editing, Resources.

## Footnotes

^1^ http://www.clsi.org

^2^ https://github.com/cjfields/readfq

^3^ http://www.pasteur.fr/mlst

## References

[ref1] Administration, U.S.F.a.D. (2009). Guidance for industry and FDA. Class II special controls guidance document: antimicrobial susceptibility test (AST) systems, U.S.F.A.D.a. Center for Devices and Radiological Health. (U.S. Department of Health and Human Services, Silver Spring, MD).

[ref2] Antimicrobial Resistance C. (2022). Global burden of bacterial antimicrobial resistance in 2019: a systematic analysis. Lancet 399, 629–655. doi: 10.1016/S0140-6736(21)02724-0, PMID: 35065702 PMC8841637

[ref3] Avershina E. Khezri A. Ahmad R. (2023). Clinical diagnostics of bacterial infections and their Resistance to antibiotics-current state and whole genome sequencing implementation perspectives. Antibiotics (Basel) 12:781. doi: 10.3390/antibiotics12040781, PMID: 37107143 PMC10135054

[ref4] Avershina E. Sharma P. Taxt A. M. Singh H. Frye S. A. Paul K.. (2021). AMR-Diag: neural network based genotype-to-phenotype prediction of resistance towards beta-lactams in Escherichia coli and *Klebsiella pneumoniae*. Comput. Struct. Biotechnol. J. 19, 1896–1906. doi: 10.1016/j.csbj.2021.03.027, PMID: 33897984 PMC8060595

[ref5] Bradley P. Gordon N. C. Walker T. M. Dunn L. Heys S. Huang B.. (2015). Rapid antibiotic-resistance predictions from genome sequence data for Staphylococcus aureus and *Mycobacterium tuberculosis*. Nat. Commun. 6:10063. doi: 10.1038/ncomms10063, PMID: 26686880 PMC4703848

[ref6] Breiman L. (2001). Random forests. Mach. Learn. 45, 5–32. doi: 10.1023/a:1010933404324

[ref7] Chen T. Guestrin C. Assoc Comp M. (2016). XGBoost: a scalable tree boosting system. In 22nd ACM SIGKDD International Conference on Knowledge Discovery and Data Mining (KDD).

[ref8] Chen Y. Ji J. Ying C. Liu Z. Yang Q. Kong H.. (2022). Blood bacterial resistant investigation collaborative system (BRICS) report: a national surveillance in China from 2014 to 2019. Antimicrob. Resist. Infect. Control 11:17. doi: 10.1186/s13756-022-01055-5, PMID: 35074014 PMC8785473

[ref9] Chen C. H. Wu P. H. Lu M. C. Ho M. W. Hsueh P. R. (2023). Geographic patterns of carbapenem-resistant, multi-drug-resistant and difficult-to-treat *Acinetobacter baumannii* in the Asia-Pacific region: results from the Antimicrobial testing leadership and surveillance (ATLAS) program, 2020. Int. J. Antimicrob. Agents 61:106707. doi: 10.1016/j.ijantimicag.2022.106707 36608719

[ref10] Chen S. Zhou Y. Chen Y. Gu J. (2018). Fastp: an ultra-fast all-in-one FASTQ preprocessor. Bioinformatics 34, i884–i890. doi: 10.1093/bioinformatics/bty560, PMID: 30423086 PMC6129281

[ref11] Cooper A. L. Low A. J. Koziol A. G. Thomas M. C. Leclair D. Tamber S.. (2020). Systematic evaluation of whole genome sequence-based predictions of Salmonella serotype and Antimicrobial Resistance. Front. Microbiol. 11:549. doi: 10.3389/fmicb.2020.00549, PMID: 32318038 PMC7147080

[ref12] Cortes C. Vapnik V. (1995). SUPPORT-VECTOR NETWORKS. Mach. Learn. 20, 273–297. doi: 10.1023/a:1022627411411

[ref13] De Oliveira D. M. P. Forde B. M. Kidd T. J. Harris P. N. A. Schembri M. A. Beatson S. A.. (2020). Antimicrobial Resistance in ESKAPE pathogens. Clin. Microbiol. Rev. 33:e00181-19. doi: 10.1128/CMR.00181-19, PMID: 32404435 PMC7227449

[ref14] Drouin A. Giguere S. Deraspe M. Marchand M. Tyers M. Loo V. G.. (2016). Predictive computational phenotyping and biomarker discovery using reference-free genome comparisons. BMC Genomics 17:754. doi: 10.1186/s12864-016-2889-6, PMID: 27671088 PMC5037627

[ref15] Eyre D. W. De Silva D. Cole K. Peters J. Cole M. J. Grad Y. H.. (2017). WGS to predict antibiotic MICs for *Neisseria gonorrhoeae*. J. Antimicrob. Chemother. 72, 1937–1947. doi: 10.1093/jac/dkx067, PMID: 28333355 PMC5890716

[ref16] Fawcett T. (2006). An introduction to ROC analysis. Pattern Recogn. Lett. 27, 861–874. doi: 10.1016/j.patrec.2005.10.010

[ref17] Hamidian M. Nigro S. J. (2019). Emergence, molecular mechanisms and global spread of carbapenem-resistant *Acinetobacter baumannii*. Microb. Genom. 5:e000306. doi: 10.1099/mgen.0.000306, PMID: 31599224 PMC6861865

[ref18] Hernandez-Gonzalez I. L. Mateo-Estrada V. Castillo-Ramirez S. (2022). The promiscuous and highly mobile resistome of *Acinetobacter baumannii*. Microb. Genom. 8:000762. doi: 10.1099/mgen.0.000762, PMID: 35075990 PMC8914355

[ref19] Hu X. Zhao Y. Han P. Liu S. Liu W. Mai C.. (2023). Novel clinical mNGS-based machine learning model for rapid Antimicrobial susceptibility testing of *Acinetobacter baumannii*. J. Clin. Microbiol. 61:e0180522. doi: 10.1128/jcm.01805-22, PMID: 37022167 PMC10204632

[ref20] Humphries R. M. Bragin E. Parkhill J. Morales G. Schmitz J. E. Rhodes P. A. (2023). Machine-learning model for prediction of Cefepime susceptibility in *Escherichia coli* from whole-genome sequencing data. J. Clin. Microbiol. 61:e0143122. doi: 10.1128/jcm.01431-22, PMID: 36840604 PMC10035297

[ref21] Jo J. Ko K. S. (2021). Tigecycline Heteroresistance and Resistance mechanism in clinical isolates of *Acinetobacter baumannii*. Microbiol. Spectr. 9:e0101021. doi: 10.1128/Spectrum.01010-21, PMID: 34523993 PMC8557860

[ref22] Kokot M. Dlugosz M. Deorowicz S. (2017). KMC 3: counting and manipulating k-mer statistics. Bioinformatics 33, 2759–2761. doi: 10.1093/bioinformatics/btx304, PMID: 28472236

[ref23] Ku Y. H. Yu W. L. (2021). Cefoperazone/sulbactam: new composites against multiresistant gram negative bacteria? Infect. Genet. Evol. 88:104707. doi: 10.1016/j.meegid.2021.104707, PMID: 33418147

[ref24] Li J. Li Y. Cao X. Zheng J. Zhang Y. Xie H.. (2023). Genome-wide identification and oxacillinase OXA distribution characteristics of Acinetobacter spp. based on a global database. Front. Microbiol. 14:1174200. doi: 10.3389/fmicb.2023.1174200, PMID: 37323896 PMC10267304

[ref25] Liu C. Chen K. Wu Y. Huang L. Fang Y. Lu J.. (2022). Epidemiological and genetic characteristics of clinical carbapenem-resistant *Acinetobacter baumannii* strains collected countrywide from hospital intensive care units (ICUs) in China. Emerg. Microbes Infect. 11, 1730–1741. doi: 10.1080/22221751.2022.2093134, PMID: 35730377 PMC9258068

[ref26] Liu Y. Wang Q. Zhao C. Chen H. Li H. Wang H.. (2020). Prospective multi-center evaluation on risk factors, clinical characteristics and outcomes due to carbapenem resistance in *Acinetobacter baumannii* complex bacteraemia: experience from the Chinese Antimicrobial Resistance surveillance of nosocomial infections (CARES) Network. J. Med. Microbiol. 69, 949–959. doi: 10.1099/jmm.0.001222, PMID: 32584215

[ref27] Macesic N. Bear O. J. Pe’er I. Tatonetti N. P. Peleg A. Y. Uhlemann A.-C. (2020). Predicting phenotypic Polymyxin Resistance in *Klebsiella pneumoniae* through machine learning analysis of genomic data. mSystems 5:e00656-19. doi: 10.1128/mSystems.00656-19, PMID: 32457240 PMC7253370

[ref28] Maguire F. Rehman M. A. Carrillo C. Diarra M. S. Beiko R. G. (2019). Identification of primary Antimicrobial Resistance drivers in agricultural Nontyphoidal *Salmonella enterica* Serovars by using machine learning. mSystems 4:e00211-19. doi: 10.1128/mSystems.00211-19, PMID: 31387929 PMC6687941

[ref29] McArthur A. G. Waglechner N. Nizam F. Yan A. Azad M. A. Baylay A. J.. (2013). The comprehensive antibiotic resistance database. Antimicrob. Agents Chemother. 57, 3348–3357. doi: 10.1128/AAC.00419-13, PMID: 23650175 PMC3697360

[ref30] Mikheenko A. Prjibelski A. Saveliev V. Antipov D. Gurevich A. (2018). Versatile genome assembly evaluation with QUAST-LG. Bioinformatics 34, i142–i150. doi: 10.1093/bioinformatics/bty266, PMID: 29949969 PMC6022658

[ref31] Nguyen M. Brettin T. Long S. W. Musser J. M. Olsen R. J. Olson R.. (2018). Developing an in silico minimum inhibitory concentration panel test for *Klebsiella pneumoniae*. Sci. Rep. 8:421. doi: 10.1038/s41598-017-18972-w 29323230 PMC5765115

[ref32] Nguyen M. Long S. W. McDermott P. F. Olsen R. J. Olson R. Stevens R. L.. (2019). Using machine learning to predict Antimicrobial MICs and associated genomic features for Nontyphoidal Salmonella. J. Clin. Microbiol. 57:e01260-18. doi: 10.1128/JCM.01260-18, PMID: 30333126 PMC6355527

[ref33] Nguyen Q. H. Ngo H. H. Nguyen-Vo T. H. Do T. T. T. Rahardja S. Nguyen B. P. (2023). eMIC-AntiKP: estimating minimum inhibitory concentrations of antibiotics towards *Klebsiella pneumoniae* using deep learning. Comput. Struct. Biotechnol. J. 21, 751–757. doi: 10.1016/j.csbj.2022.12.041, PMID: 36659924 PMC9827358

[ref34] Nguyen M. Olson R. Shukla M. VanOeffelen M. Davis J. J. (2020). Predicting antimicrobial resistance using conserved genes. PLoS Comput. Biol. 16:e1008319. doi: 10.1371/journal.pcbi.1008319, PMID: 33075053 PMC7595632

[ref35] Opota O. Croxatto A. Prod'hom G. Greub G. (2015). Blood culture-based diagnosis of bacteraemia: state of the art. Clin. Microbiol. Infect. 21, 313–322. doi: 10.1016/j.cmi.2015.01.003 25753137

[ref36] Pedregosa F. Varoquaux G. Gramfort A. Michel V. Thirion B. Grisel O.. (2011). Scikit-learn: machine learning in Python. J. Mach. Learn. Res. 12, 2825–2830. Available at: https://jmlr.csail.mit.edu/papers/v12/pedregosa11a.html

[ref37] Pesesky M. W. Hussain T. Wallace M. Patel S. Andleeb S. Burnham C. D.. (2016). Evaluation of machine learning and rules-based approaches for predicting Antimicrobial Resistance profiles in gram-negative Bacilli from whole genome sequence data. Front. Microbiol. 7:1887. doi: 10.3389/fmicb.2016.01887, PMID: 27965630 PMC5124574

[ref38] Porse A. Jahn L. J. Ellabaan M. M. H. Sommer M. O. A. (2020). Dominant resistance and negative epistasis can limit the co-selection of de novo resistance mutations and antibiotic resistance genes. Nat. Commun. 11:1199. doi: 10.1038/s41467-020-15080-8, PMID: 32139686 PMC7057998

[ref39] Prjibelski A. Antipov D. Meleshko D. Lapidus A. Korobeynikov A. (2020). Using SPAdes De Novo Assembler. Curr. Protoc. Bioinformatics 70:e102. doi: 10.1002/cpbi.102 32559359

[ref40] Rice L. B. (2008). Federal funding for the study of antimicrobial resistance in nosocomial pathogens: no ESKAPE. J. Infect. Dis. 197, 1079–1081. doi: 10.1086/533452, PMID: 18419525

[ref41] Rodrigues D. L. N. Morais-Rodrigues F. Hurtado R. Dos Santos R. G. Costa D. C. Barh D.. (2021). Pan-Resistome insights into the multidrug Resistance of *Acinetobacter baumannii*. Antibiotics (Basel) 10:596. doi: 10.3390/antibiotics10050596, PMID: 34069870 PMC8157372

[ref42] Stoesser N. Batty E. M. Eyre D. W. Morgan M. Wyllie D. H. Del Ojo Elias C.. (2013). Predicting antimicrobial susceptibilities for Escherichia coli and *Klebsiella pneumoniae* isolates using whole genomic sequence data. J. Antimicrob. Chemother. 68, 2234–2244. doi: 10.1093/jac/dkt180, PMID: 23722448 PMC3772739

[ref43] Sunuwar J. Azad R. K. (2021). A machine learning framework to predict antibiotic resistance traits and yet unknown genes underlying resistance to specific antibiotics in bacterial strains. Brief. Bioinform. 22:bbab179. doi: 10.1093/bib/bbab179, PMID: 34015806

[ref44] Vogwill T. Kojadinovic M. MacLean R. C. (2016). Epistasis between antibiotic resistance mutations and genetic background shape the fitness effect of resistance across species of Pseudomonas. Proc. Biol. Sci. 283:20160151. doi: 10.1098/rspb.2016.0151, PMID: 27170722 PMC4874708

[ref45] Wang C. C. Hung Y. T. Chou C. Y. Hsuan S. L. Chen Z. W. Chang P. Y.. (2023). Using random forest to predict antimicrobial minimum inhibitory concentrations of nontyphoidal Salmonella in Taiwan. Vet. Res. 54:11. doi: 10.1186/s13567-023-01141-5, PMID: 36747286 PMC9903507

[ref46] Wattam A. R. Davis J. J. Assaf R. Boisvert S. Brettin T. Bun C.. (2017). Improvements to PATRIC, the all-bacterial bioinformatics database and analysis resource center. Nucleic Acids Res. 45, D535–D542. doi: 10.1093/nar/gkw1017, PMID: 27899627 PMC5210524

[ref47] Wei C. Chen J. Anwar T. M. Huang L. Yang W. Dong X.. (2023). Genomic determinants of pathogenicity and Antimicrobial Resistance of nosocomial *Acinetobacter baumannii* clinical isolates of hospitalized patients (2019-2021) from a sentinel Hospital in Hangzhou, China. Infect. Drug Resist. 16, 2939–2952. doi: 10.2147/IDR.S407577, PMID: 37201122 PMC10187652

[ref48] Wes M. (2011). Pandas: A foundational Python library for data analysis and statistics. Python High Performance Science Computer. Available at: https://www.researchgate.net/publication/265194455_pandas_a_Foundational_Python_Library_for_Data_Analysis_and_Statistics

[ref49] Yan M. Zheng B. Li Y. Lv Y. (2022). Antimicrobial susceptibility trends among gram-negative Bacilli causing bloodstream infections: results from the China Antimicrobial Resistance surveillance trial (CARST) program, 2011-2020. Infect. Drug Resist. 15, 2325–2337. doi: 10.2147/IDR.S358788, PMID: 35517902 PMC9064452

[ref50] Yin Y. Zhao C. Li H. Jin L. Wang Q. Wang R.. (2021). Clinical and microbiological characteristics of adults with hospital-acquired pneumonia: a 10-year prospective observational study in China. Eur. J. Clin. Microbiol. Infect. Dis. 40, 683–690. doi: 10.1007/s10096-020-04046-9, PMID: 33029764 PMC7540435

[ref51] Yu K. Zeng W. Xu Y. Liao W. Xu W. Zhou T.. (2021). Bloodstream infections caused by ST2 *Acinetobacter baumannii*: risk factors, antibiotic regimens, and virulence over 6 years period in China. Antimicrob. Resist. Infect. Control 10:16. doi: 10.1186/s13756-020-00876-6, PMID: 33461617 PMC7814448

[ref52] Zankari E. Hasman H. Cosentino S. Vestergaard M. Rasmussen S. Lund O.. (2012). Identification of acquired antimicrobial resistance genes. J. Antimicrob. Chemother. 67, 2640–2644. doi: 10.1093/jac/dks261, PMID: 22782487 PMC3468078

