# Supplementary material for: Machine learning and feature extraction for rapid antimicrobial resistance prediction of Acinetobacter baumannii from whole-genome sequencing data
Source: Front Microbiol. 2024 Jan 11;14:1320312. doi: 10.3389/fmicb.2023.1320312 (PMC10808480; doi:10.3389/fmicb.2023.1320312)
Supplement: Supplementary file 1 [file Data_Sheet_1.docx]

**Supplemental Materials**

**Figure S1. The ROC curves (receiver operating characteristic curve) and the AUC (Area**

**Under Curve) value for IPM.**


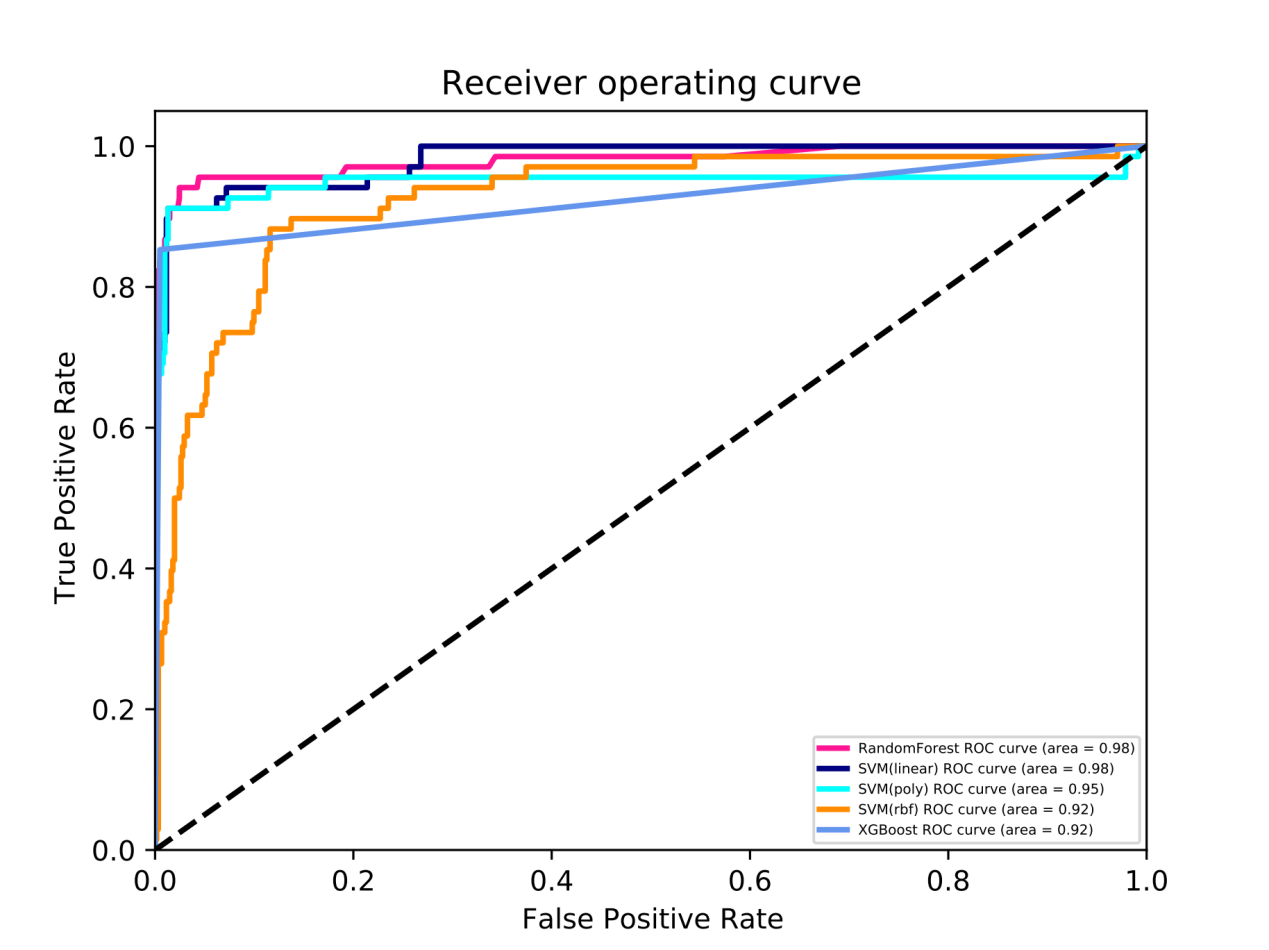


**Figure S2. The ROC curves (receiver operating characteristic curve) and the AUC (Area**

**Under Curve) value for MEM.**


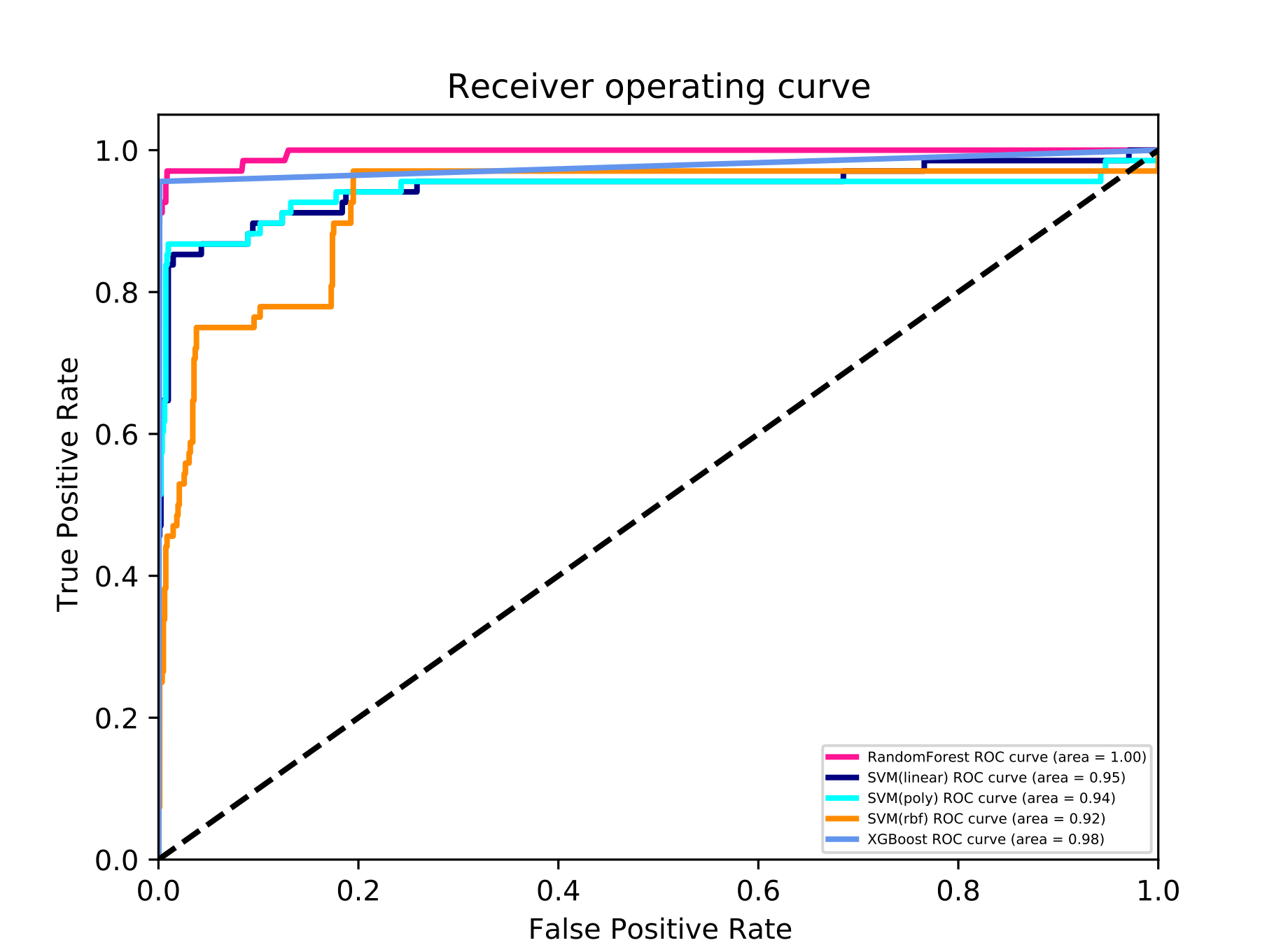


**Figure S3. The ROC curves (receiver operating characteristic curve) and the AUC (Area**

**Under Curve) value for CST.**


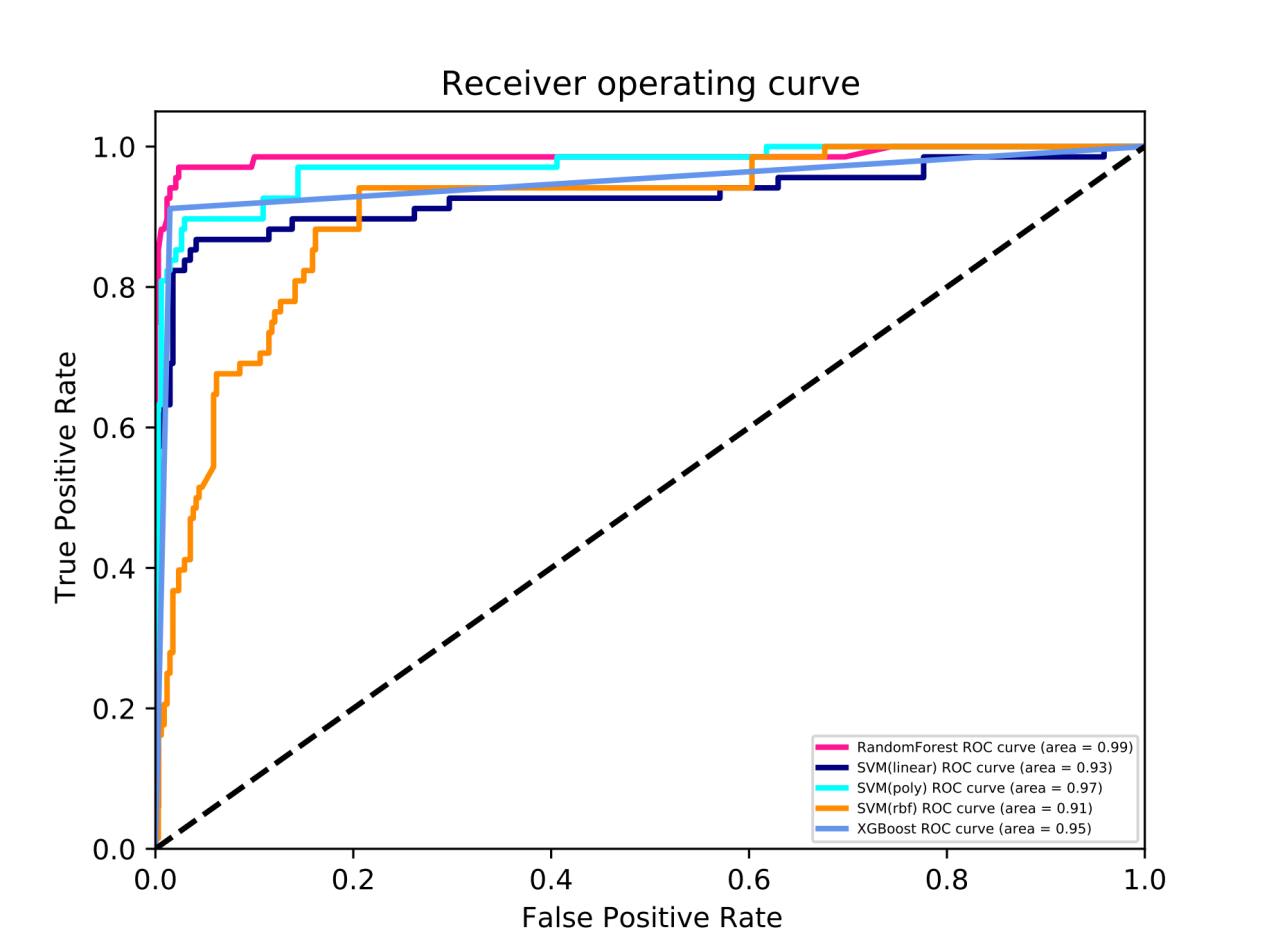


**Figure S4. The ROC curves (receiver operating characteristic curve) and the AUC (Area**

**Under Curve) value for TGC.**


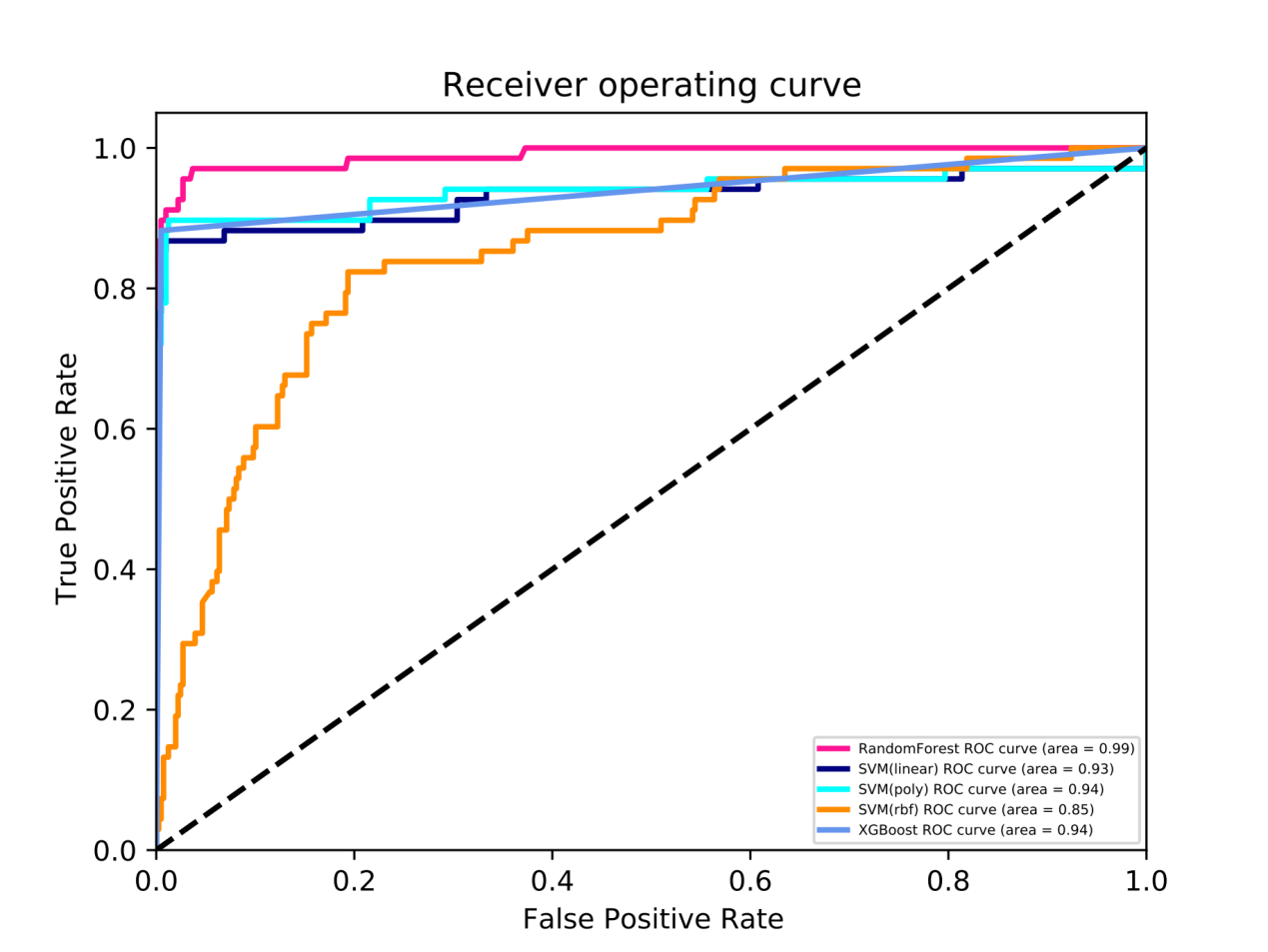


**Figure S5. The ROC curves (receiver operating characteristic curve) and the AUC (Area**

**Under Curve) value for FEP.**


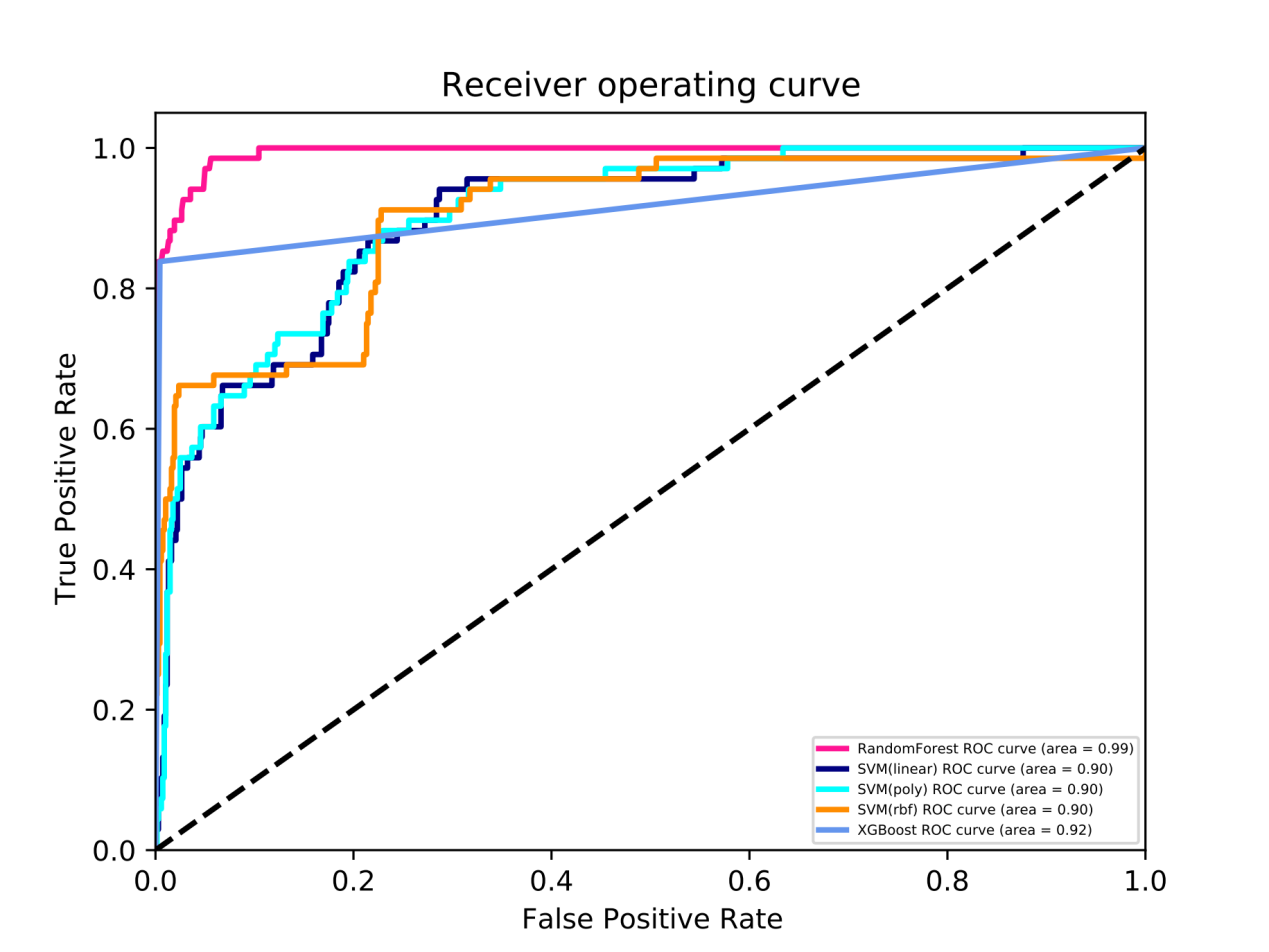


**Figure S6. The ROC curves (receiver operating characteristic curve) and the AUC (Area**

**Under Curve) value for CAZ.**


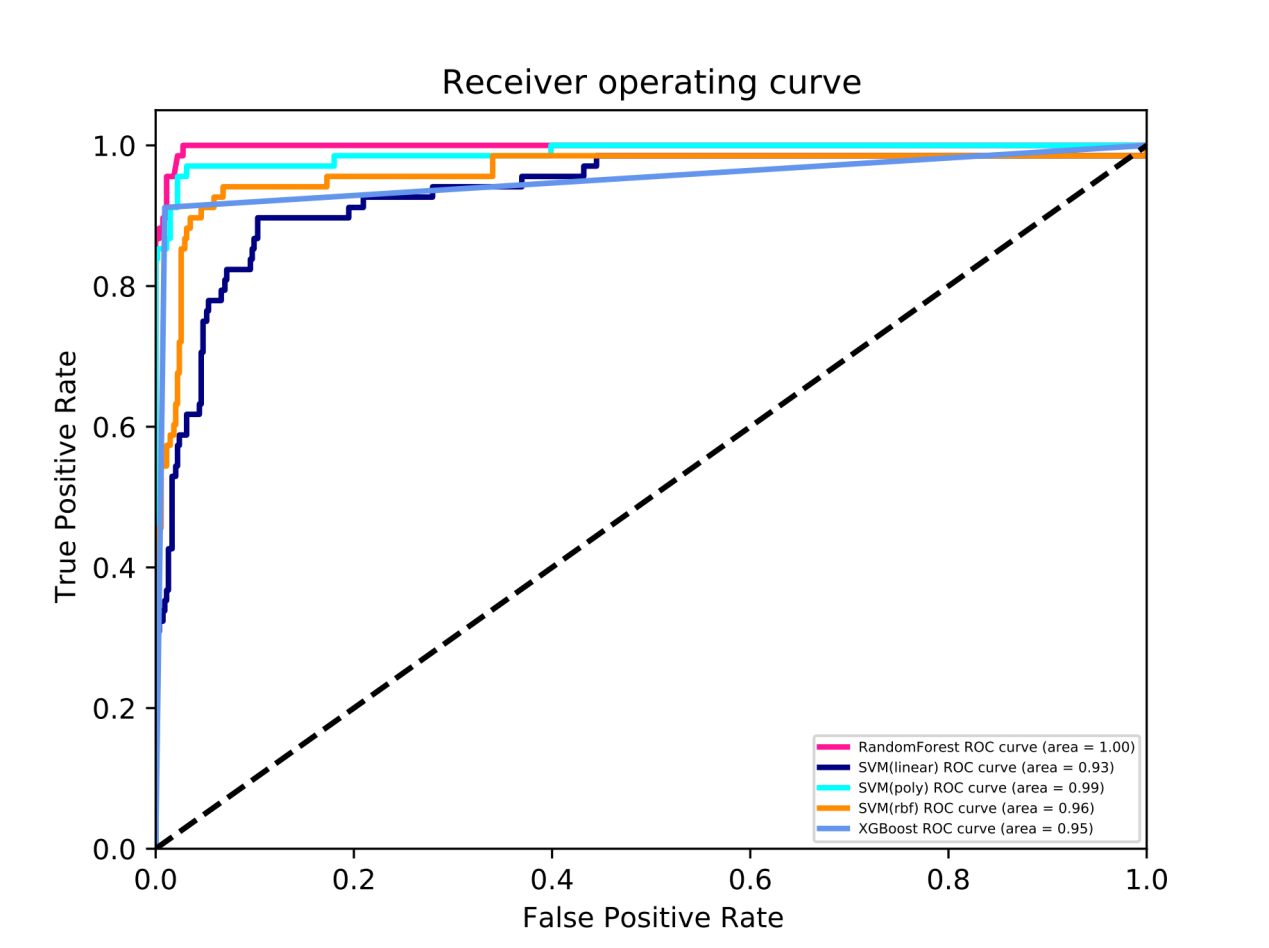


**Figure S7. The ROC curves (receiver operating characteristic curve) and the AUC (Area**

**Under Curve) value for CSL.**


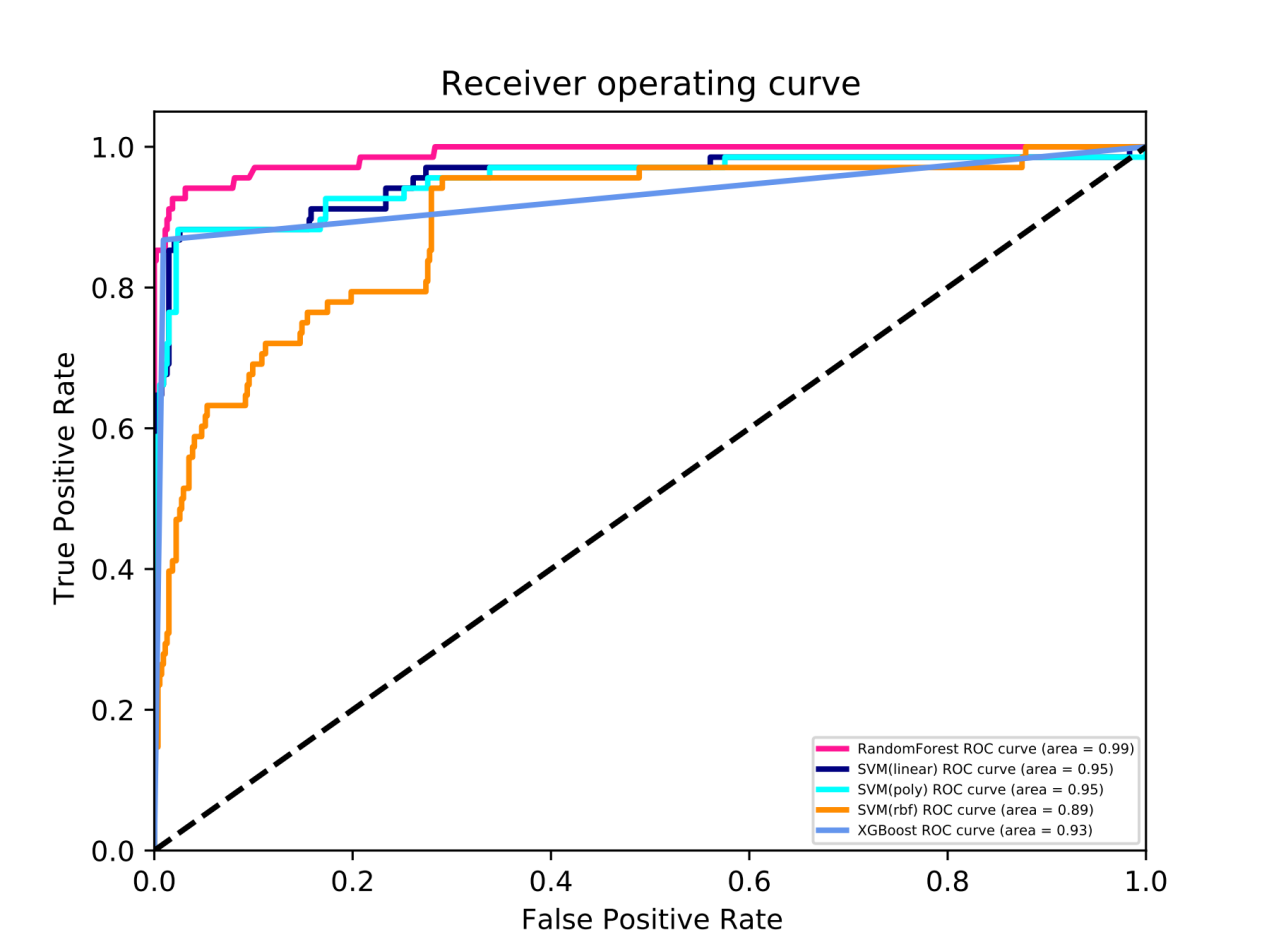


**Figure S8. The ROC curves (receiver operating characteristic curve) and the AUC (Area**

**Under Curve) value for TZP.**


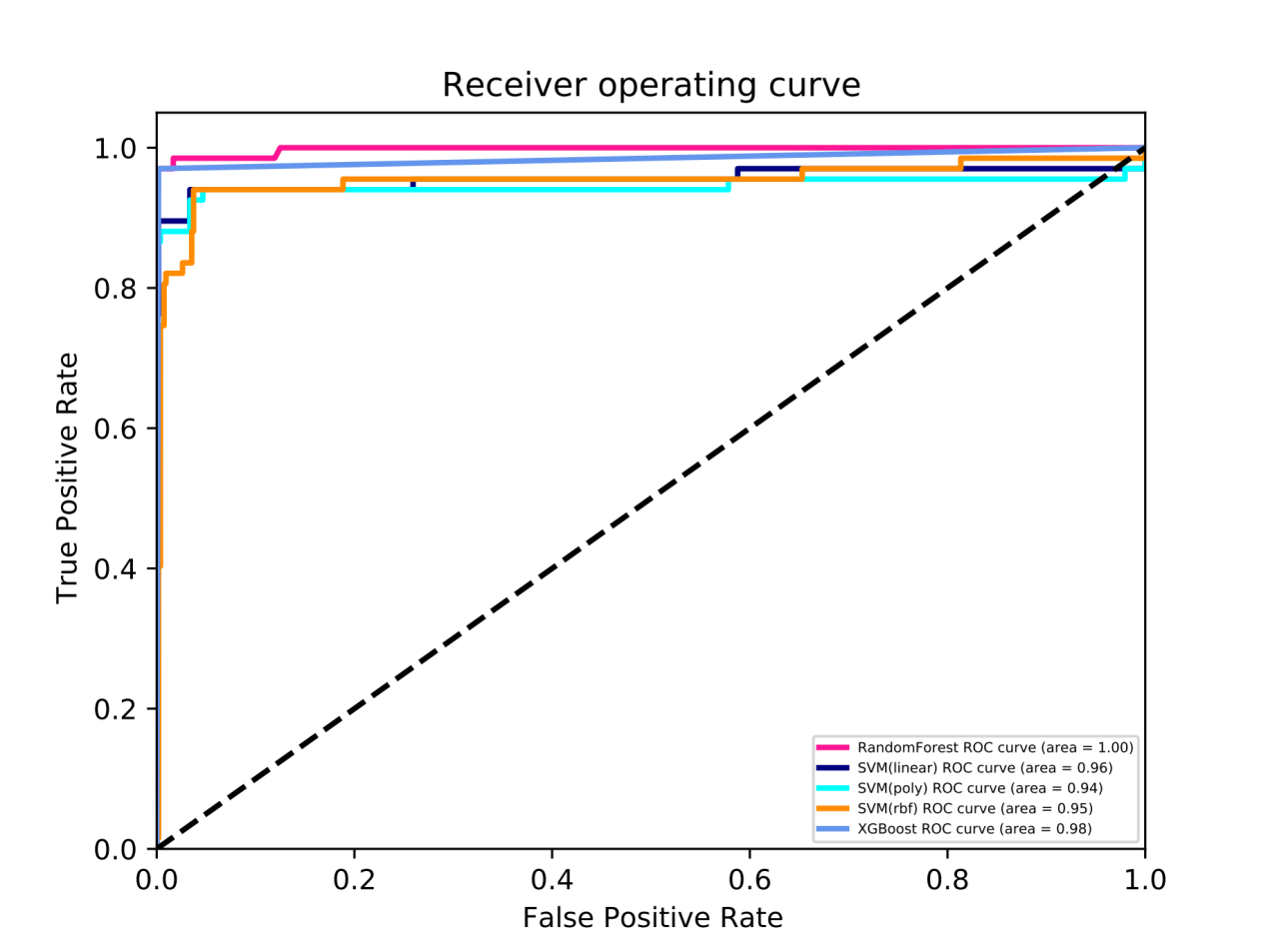


**Figure S9. The ROC curves (receiver operating characteristic curve) and the AUC (Area Under Curve) value for AMK.**


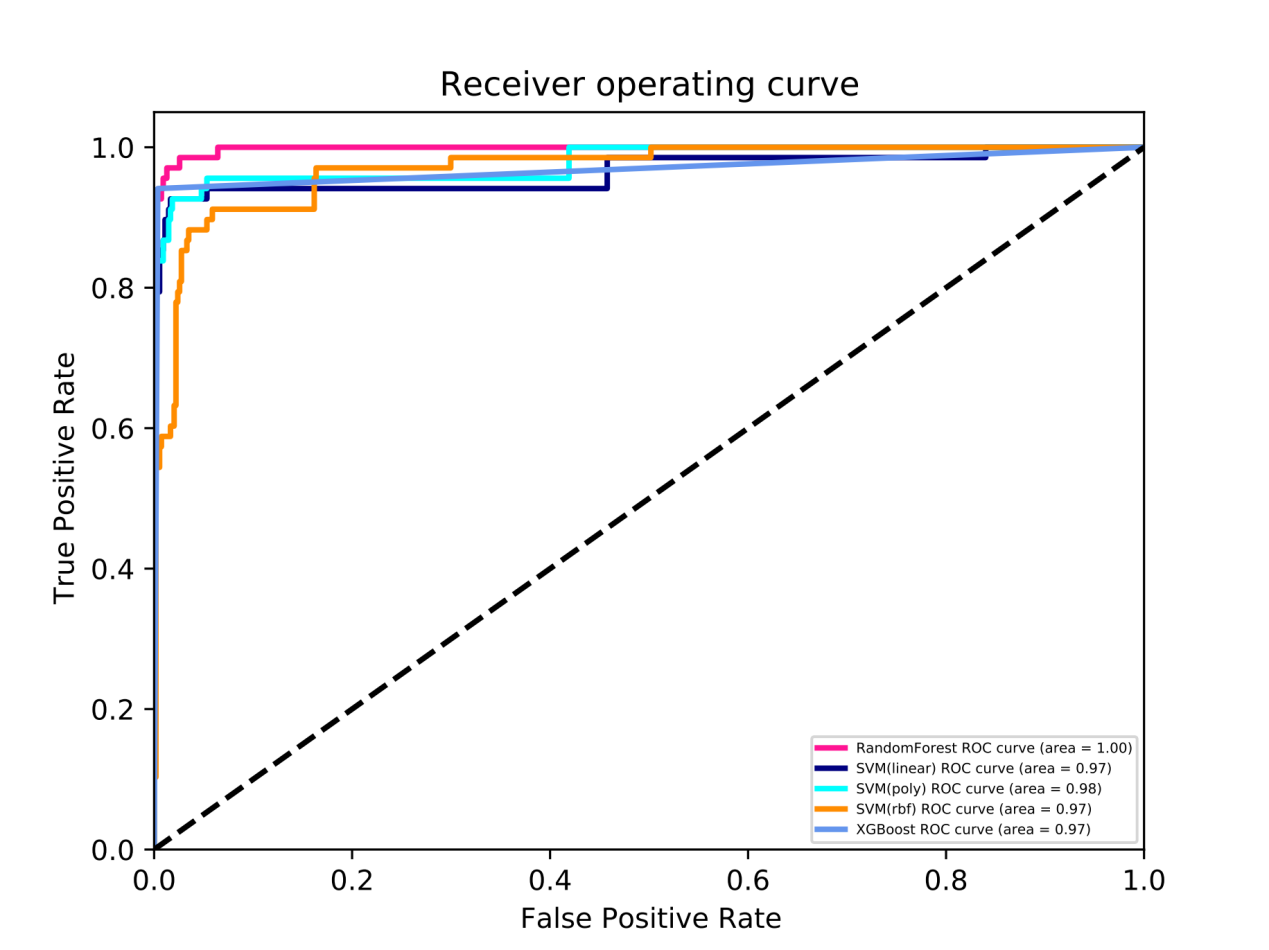


**Figure S10. The ROC curves (receiver operating characteristic curve) and the AUC (Area**

**Under Curve) value for CIP.**


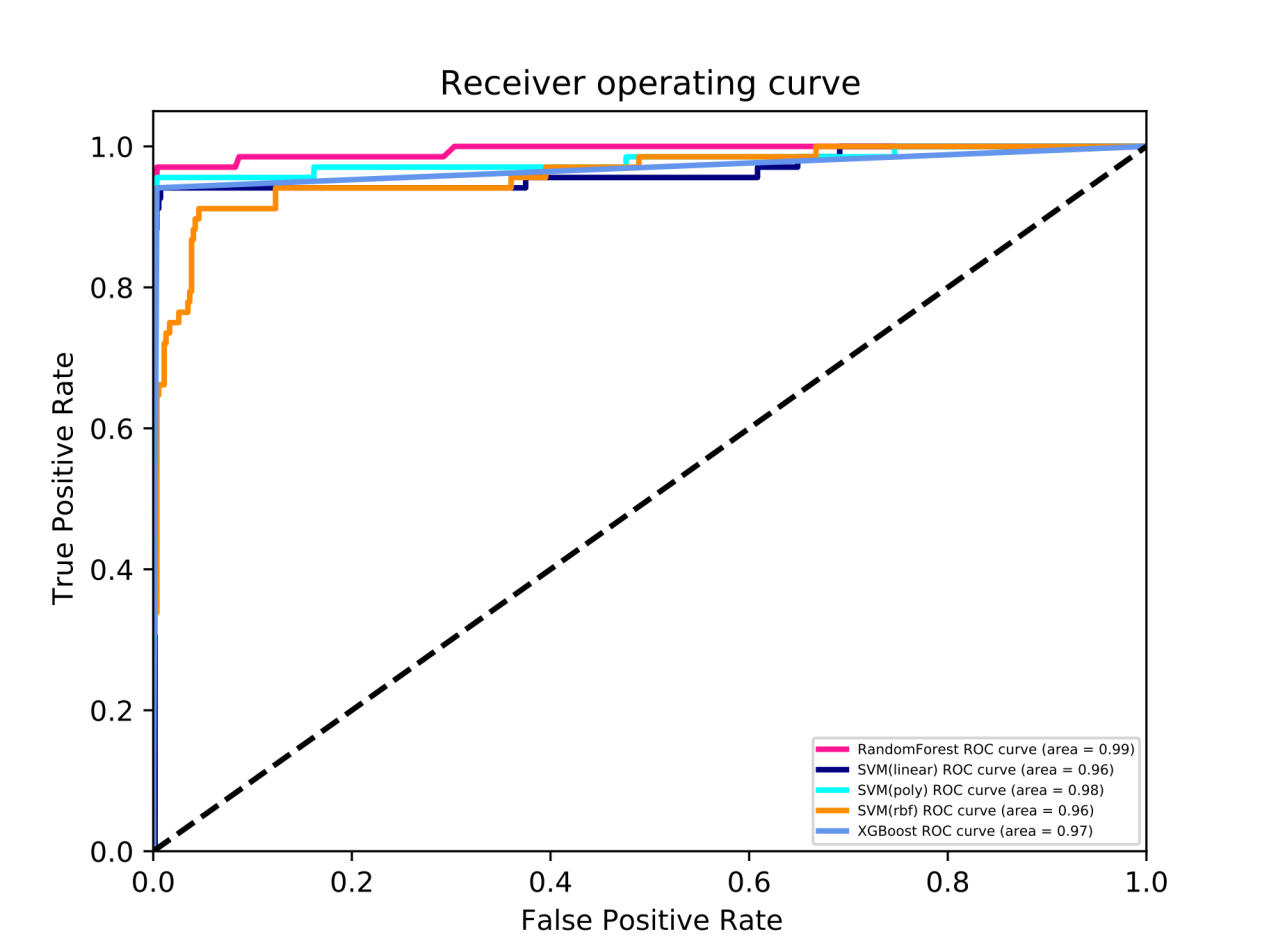


**Figure S11. The ROC curves (receiver operating characteristic curve) and the AUC (Area**

**Under Curve) value for LVX.**


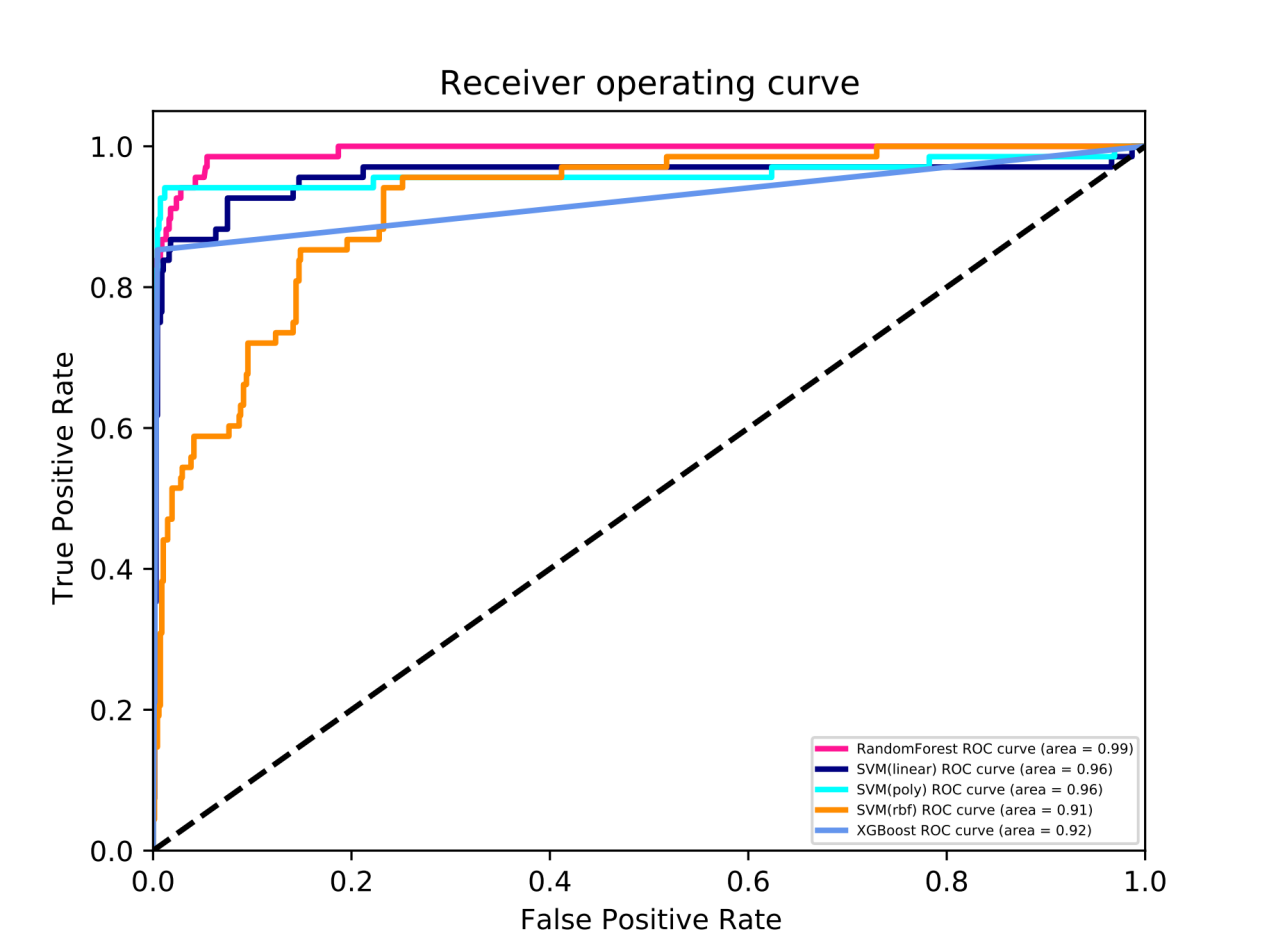


**Figure S12. The ROC curves (receiver operating characteristic curve) and the AUC (Area**

**Under Curve) value for MIN.**


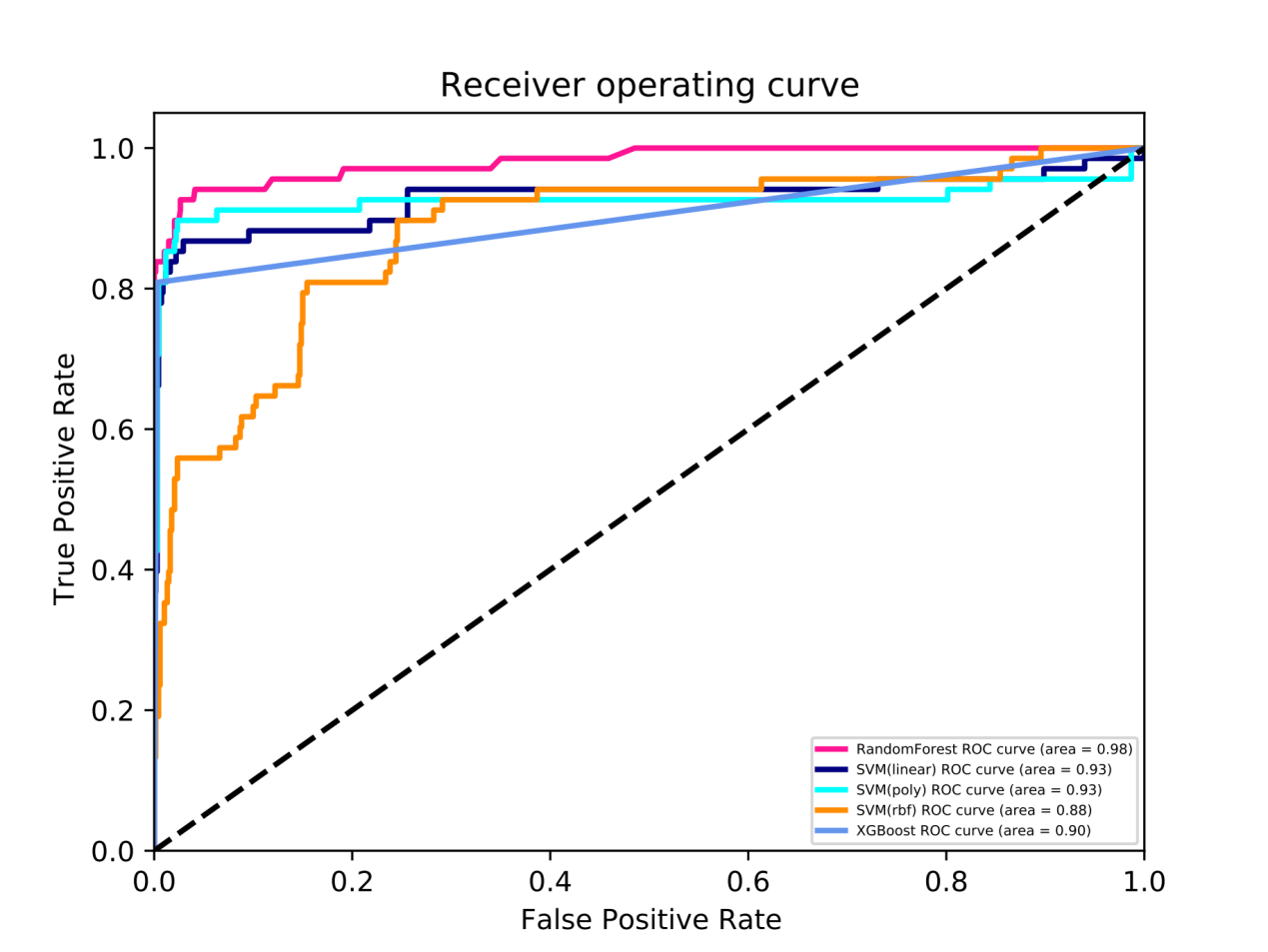


**Figure S13. The ROC curves (receiver operating characteristic curve) and the AUC (Area**

**Under Curve) value for SXT.**


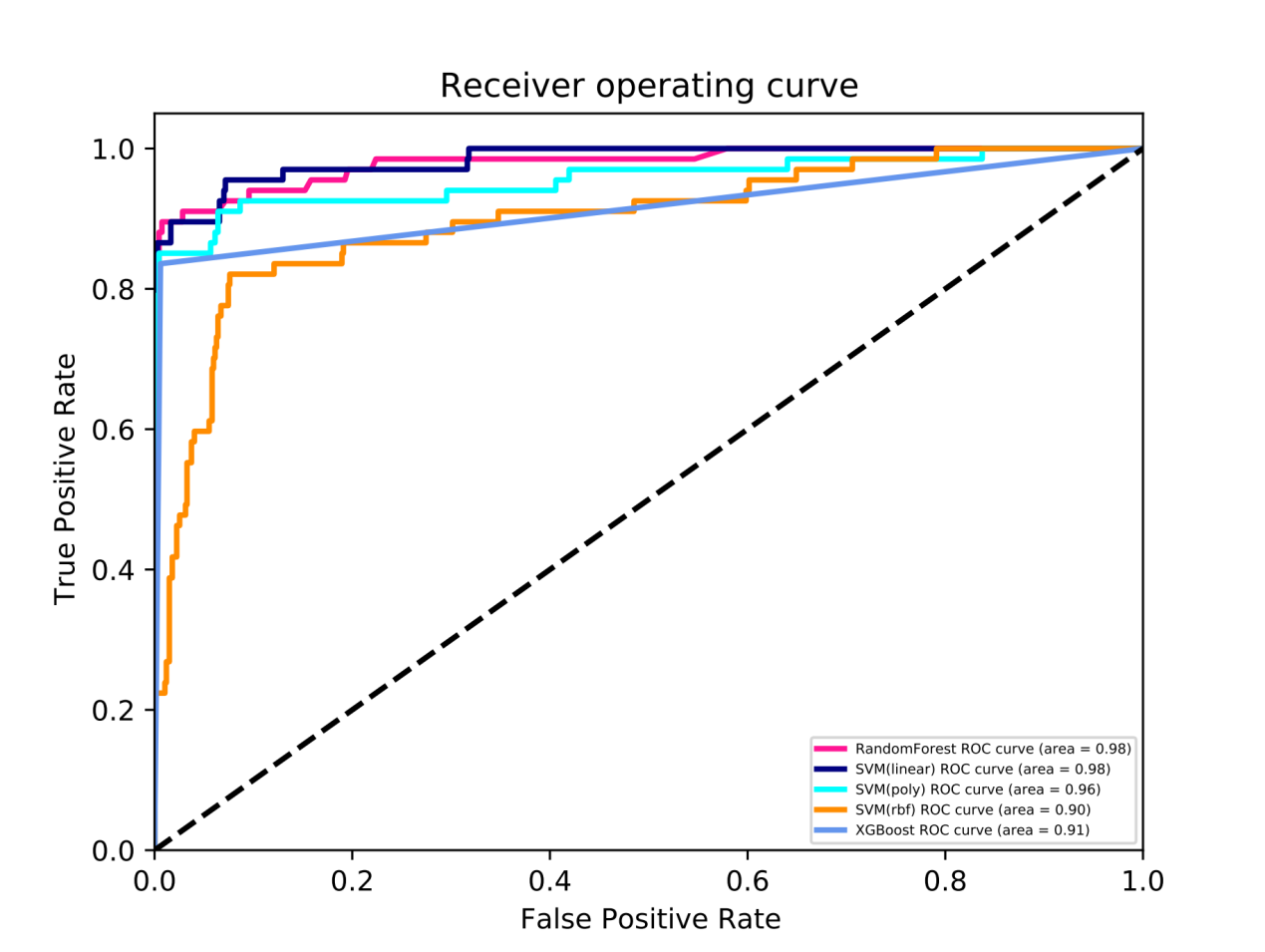


**Table S4. The average AUC values and 95% confidence interval (CI) of the cross-validation results for all five models.**

**Table S5. Prediction of the minimum inhibitory concentration (MIC) for IPM.** Horizontal axis are the prediction results and the vertical axis are the standard results.

**Table S6. Prediction of the susceptible (S), intermediate (I) and resistant (R) categories for IPM.** Horizontal axis are the prediction results and the vertical axis are the standard results.

**Table S7. Prediction of the minimum inhibitory concentration (MIC) for MEM.** Horizontal axis are the prediction results and the vertical axis are the standard results.

**Table S8. Prediction of the susceptible (S), intermediate (I) and resistant (R) categories for MEM.** Horizontal axis are the prediction results and the vertical axis are the standard results.

**Table S9. Prediction of the minimum inhibitory concentration (MIC) for CST.** Horizontal axis are the prediction results and the vertical axis are the standard results.

**Table S10. Prediction of the susceptible (S) and intermediate (I) categories for CST.** Horizontal axis are the prediction results and the vertical axis are the standard results.

**Table S11. Prediction of the minimum inhibitory concentration (MIC) for TGC.**

Horizontal axis are the prediction results and the vertical axis are the standard results.

**Table S12. Prediction of the susceptible (S), intermediate (I) and resistant (R) categories for TGC.** Horizontal axis are the prediction results and the vertical axis are the standard results.

**Table S13. Prediction of the minimum inhibitory concentration (MIC) for FEP.** Horizontal axis are the prediction results and the vertical axis are the standard results.

**Table S14. Prediction of the susceptible (S) and resistant (R) categories for FEP.** Horizontal axis are the prediction results and the vertical axis are the standard results.

**Table S15. Prediction of the minimum inhibitory concentration (MIC) for CAZ.** Horizontal axis are the prediction results and the vertical axis are the standard results.

**Table S16. Prediction of the susceptible (S), intermediate (I) and resistant (R) categories for CAZ.** Horizontal axis are the prediction results and the vertical axis are the standard results.

**Table S17. Prediction of the minimum inhibitory concentration (MIC) for CSL.** Horizontal axis are the prediction results and the vertical axis are the standard results.

**Table S18. Prediction of the susceptible (S) and resistant (R) categories for CSL.** Horizontal axis are the prediction results and the vertical axis are the standard results.

**Table S19. Prediction of the minimum inhibitory concentration (MIC) for TZP.** Horizontal axis are the prediction results and the vertical axis are the standard results.

**Table S20. Prediction of the susceptible (S) and resistant (R) categories for TZP.** Horizontal axis are the prediction results and the vertical axis are the standard results.

**Table S21. Prediction of the minimum inhibitory concentration (MIC) for AMK.** Horizontal axis are the prediction results and the vertical axis are the standard results.

**Table S22. Prediction of the susceptible (S), intermediate (I) and resistant (R) categories for AMK.** Horizontal axis are the prediction results and the vertical axis are the standard results.

**Table S23. Prediction of the minimum inhibitory concentration (MIC) for CIP.** Horizontal axis are the prediction results and the vertical axis are the standard results.

**Table S24. Prediction of the susceptible (S) and resistant (R) categories for CIP.** Horizontal axis are the prediction results and the vertical axis are the standard results.

**Table S25. Prediction of the minimum inhibitory concentration (MIC) for LVX.** Horizontal axis are the prediction results and the vertical axis are the standard results.

**Table S26. Prediction of the susceptible (S), intermediate (I) and resistant (R) categories for LVX.** Horizontal axis are the prediction results and the vertical axis are the standard results.

**Table S27. Prediction of the minimum inhibitory concentration (MIC) for MIN.** Horizontal axis are the prediction results and the vertical axis are the standard results.

**Table S28. Prediction of the susceptible (S), intermediate (I) and resistant (R) categories for MIN.** Horizontal axis are the prediction results and the vertical axis are the standard results.

**Table S29. Prediction of the minimum inhibitory concentration (MIC) for SXT.** Horizontal axis are the prediction results and the vertical axis are the standard results.

**Table S30. Prediction of the susceptible (S) and resistant (R) categories for SXT.** Horizontal axis are the prediction results and the vertical axis are the standard results.
